# EEG Data Quality: Determinants and Impact in a Multicenter Study of Children, Adolescents, and Adults with Attention-Deficit/Hyperactivity Disorder (ADHD)

**DOI:** 10.3390/brainsci11020214

**Published:** 2021-02-10

**Authors:** Anna Kaiser, Pascal-M. Aggensteiner, Martin Holtmann, Andreas Fallgatter, Marcel Romanos, Karina Abenova, Barbara Alm, Katja Becker, Manfred Döpfner, Thomas Ethofer, Christine M. Freitag, Julia Geissler, Johannes Hebebrand, Michael Huss, Thomas Jans, Lea Teresa Jendreizik, Johanna Ketter, Tanja Legenbauer, Alexandra Philipsen, Luise Poustka, Tobias Renner, Wolfgang Retz, Michael Rösler, Johannes Thome, Henrik Uebel-von Sandersleben, Elena von Wirth, Toivo Zinnow, Sarah Hohmann, Sabina Millenet, Nathalie E. Holz, Tobias Banaschewski, Daniel Brandeis, on behalf of the ESCAlife-Consortium

**Affiliations:** 1 Department of Child and Adolescent Psychiatry and Psychotherapy, Central Institute of Mental Health, Medical Faculty Mannheim/Heidelberg University, 68159 Mannheim, Germany; Pascal.Aggensteiner@zi-mannheim.de (P.-M.A.); Karina.Abenova@zi-mannheim.de (K.A.); Sarah.Hohmann@zi-mannheim.de (S.H.); Sabina.Millenet@zi-mannheim.de (S.M.); Nathalie.Holz@zi-mannheim.de (N.E.H.); tobias.banaschewski@zi-mannheim.de (T.B.); Daniel.Brandeis@zi-mannheim.de (D.B.); 2 LWL-University Hospital for Child and Adolescent Psychiatry, Psychotherapy, and Psychosomatics, Ruhr University Bochum, 59071 Hamm, Germany; martin.holtmann@lwl.org (M.H.); tanja.legenbauer@lwl.org (T.L.); 3 Tübingen University Hospital for Psychiatry and Psychotherapy, 72076 Tübingen, Germany; Andreas.Fallgatter@med.uni-tuebingen.de (A.F.); Thomas.Ethofer@med.uni-tuebingen.de (T.E.); 4 Department of Child and Adolescent Psychiatry, Psychosomatics, and Psychotherapy, Center of Mental Health, University Hospital of Würzburg, 97080 Würzburg, Germany; Romanos_M@ukw.de (M.R.); Geissler_J@ukw.de (J.G.); Jans_T@ukw.de (T.J.); 5 Department of Psychiatry and Psychotherapy, Central Institute of Mental Health, Medical Faculty Mannheim/Heidelberg University, 68159 Mannheim, Germany; Barbara.Alm@zi-mannheim.de; 6 Department of Child and Adolescent Psychiatry, Psychosomatics and Psychotherapy, Medical Faculty Philipps-University Marburg and University Hospital Marburg, 35039 Marburg, Germany; katja.becker@med.uni-marburg.de (K.B.); ketterj@med.uni-marburg.de (J.K.); 7 Center for Mind, Brain and Behavior (CMBB), University of Marburg and Justus Liebig University Giessen, 35032 Marburg, Germany; 8 Department of Child and Adolescent Psychiatry, Psychosomatics and Psychotherapy, Faculty of Medicine and University Hospital Cologne, University of Cologne, 50931 Cologne, Germany; manfred.doepfner@uk-koeln.de (M.D.); lea.jendreizik@uk-koeln.de (L.T.J.); elena.von-wirth@uk-koeln.de (E.v.W.); 9 Department of Child and Adolescent Psychiatry, Psychosomatics and Psychotherapy, University Hospital Frankfurt, Goethe University, 60323 Frankfurt am Main, Germany; C.Freitag@em.uni-frankfurt.de; 10 Department of Child and Adolescent Psychiatry, Psychosomatics and Psychotherapy, University Hospital Essen, University of Duisburg-Essen, 45147 Essen, Germany; johannes.hebebrand@lvr.de; 11 Department of Child and Adolescent Psychiatry, University Medical Center of the Johannes Gutenberg-University Mainz, 55131 Mainz, Germany; m.huss@rfk.landeskrankenhaus.de; 12 Department of Psychiatry and Psychotherapy, University of Bonn, 53127 Bonn, Germany; Alexandra.Philipsen@ukbonn.de; 13 Department of Child and Adolescent Psychiatry and Psychotherapy, University Medical Centre Göttingen, 37075 Göttingen, Germany; luise.poustka@med.uni-goettingen.de (L.P.); huebel@gwdg.de (H.U.-v.S.); 14 Department of Child and Adolescence Psychiatry and Psychotherapy, University of Tübingen, 72076 Tübingen, Germany; tobias.renner@med.uni-tuebingen.de; 15 Department of Psychiatry and Psychotherapy, University Medical Center Mainz, 55131 Mainz, Germany; wolfgang.retz@unimedizin-mainz.de; 16 Institute for Forensic Psychology and Psychiatry, Saarland University, 66424 Homburg, Germany; Michael.Roesler@uks.eu (M.R.); Toivo.Zinnow@uks.eu (T.Z.); 17 Department of Psychiatry and Psychotherapy, University Medical Center Rostock, 18055 Rostock, Germany; johannes.thome@med.uni-rostock.de; 18 Department of Child and Adolescent Psychiatry and Psychotherapy, University Hospital of Psychiatry, University of Zürich, 8032 Zürich, Switzerland; 19 Center for Integrative Human Physiology, University of Zürich, 8057 Zürich, Switzerland; 20 Neuroscience Center Zürich, Swiss Federal Institute of Technology and University of Zürich, 8057 Zürich, Switzerland

**Keywords:** electroencephalography (EEG), data quality, attention-deficit/hyperactivity disorder (ADHD), artifacts, multicenter study

## Abstract

Electroencephalography (EEG) represents a widely established method for assessing altered and typically developing brain function. However, systematic studies on EEG data quality, its correlates, and consequences are scarce. To address this research gap, the current study focused on the percentage of artifact-free segments after standard EEG pre-processing as a data quality index. We analyzed participant-related and methodological influences, and validity by replicating landmark EEG effects. Further, effects of data quality on spectral power analyses beyond participant-related characteristics were explored. EEG data from a multicenter ADHD-cohort (age range 6 to 45 years), and a non-ADHD school-age control group were analyzed (n_total_ = 305). Resting-state data during eyes open, and eyes closed conditions, and task-related data during a cued Continuous Performance Task (CPT) were collected. After pre-processing, general linear models, and stepwise regression models were fitted to the data. We found that EEG data quality was strongly related to demographic characteristics, but not to methodological factors. We were able to replicate maturational, task, and ADHD effects reported in the EEG literature, establishing a link with EEG-landmark effects. Furthermore, we showed that poor data quality significantly increases spectral power beyond effects of maturation and symptom severity. Taken together, the current results indicate that with a careful design and systematic quality control, informative large-scale multicenter trials characterizing neurophysiological mechanisms in neurodevelopmental disorders across the lifespan are feasible. Nevertheless, results are restricted to the limitations reported. Future work will clarify predictive value.

## 1. Introduction

Electroencephalography (EEG) is a non-invasive method for assessing brain-electrical activity on the scalp using a set number of electrodes [1,2]. It has been widely used in the research fields of physiology, psychology, neuroscience, and cognitive science to explore the neural dynamics and circuits related to typically developing and altered human information processing and behavior [3]. The weak surface EEG signal measured on the scalp is extremely susceptible to interferences during the process of signal collection. Significant signal distortions due to contamination through participant-induced artifacts or experimental factors sometimes lead to unavailability of sufficient EEG data for subsequent analyses, resulting in a lower reliability of study results [4]. To this end, a series of offline processing methods exists that are applied to EEG data for extracting uncontaminated signals prior to further analyses. However, there is little standardization, and pre-processing methods vary substantially [5,6].

As the quality of the raw data crucially impacts the validity of analyses and interpretation of scientific results obtained from EEG, assessments of data quality are essential. Evaluating the quality of the raw EEG signals ensures that established standards are met, and results are replicable [7]. Especially, when EEG data are recorded at multiple sites, in developmental populations, and in patient samples prone to EEG artifacts, they are characterized by a high degree of artifact contamination. For example, data from patients with attention-deficit/hyperactivity disorder (ADHD) are often contaminated by movement artifacts due to symptoms of hyperactivity. The assessment of developmental and/or psychiatric populations is typically associated with various challenges, subsequently contributing to lower EEG data quality: Children often have problems following instructions (e.g., not to move during the measurement, to pay attention to task instructions). Further, study protocols typically include far shorter measurement durations for a higher level of tolerance resulting in a lower number of data points available for final evaluations. Additionally, measures to assess physiological artifacts (such as EOG electrodes for ocular movement contamination) are often not implemented. From a data processing perspective, extracting uncontaminated signals from such EEG recordings represents a particular challenge. However, those signal distortions might provide additional useful information characterizing specific developmental and psychiatric populations. Data quality might be systematically related to age or specific psychiatric symptom dimensions with a potential relevance for classification purpose. This aspect is often neglected and not explicitly addressed in ongoing clinical trials using EEG.

Although the EEG represents an established method for assessing neuronal activity, systematic explorations of signal contamination are rather scarce and existing reports of data quality measures are often inconsistent. Typically, studies only indirectly address data quality by reporting impedance cut-offs (such as < 20 kΩ at each electrode location) or standard cut-off values for the least-acceptable absolute number of sweeps included per participant for subsequent analyses [8,9,10]. Further studies calculated analytical indices using complex models to explicitly assess EEG data quality [11,12]. However, previous reports were mainly focusing on data quality of wearable dry-electrode devices or when EEG and functional magnetic resonance imaging (fMRI) data were collected simultaneously [13]. Other recent work focused on online-monitoring of data quality for neurofeedback and brain–computer interface (BCI) applications [14]. Due to this lack of direct assessment and consistent reporting, study quality can often only be indirectly inferred from publications on EEG data. The same refers to study-specific variables potentially influencing it [15,16].

Identifying and adequately addressing EEG signal distortions ensures reliability of study results. Beyond this, replicating robust landmark effects of the EEG literature informs about the validity of analyzed data. However, to date there is little published data on such appropriate validation analyses representing replication analyses of robust landmark effects typically reported in the EEG literature that establish a link with data quality. Only few replication studies have been done so far. Nevertheless, they are urgently needed for consolidation of results in the field of EEG research [17]. Thereby, several robust landmark effects have been identified (we do not claim for a comprehensive review of all relevant EEG landmark effects): (I) For example, in the literature on resting-state EEG activity age effects of increasing fast oscillatory activity and decreasing slow oscillatory activity due to brain maturational processes have been consistently reported [18,19,20,21,22,23,24]. (II) Furthermore, a substantial amount of studies reported on alpha blocking after transition from resting state eyes closed to eyes open or task-related conditions, indicating a decrease in alpha activity primarily in occipital brain regions [25,26,27,28,29]. (III) For task-related inhibitory control activity assessed via Go/NoGo-paradigms, previous studies showed a substantially higher amplitude of the Go-P3 event-related potential (ERP) compared to the NoGo-P3, especially at posterior brain regions (e.g., [30,31]). This effect indicates a substantially stronger neurophysiological activity in response to Go- compared to NoGo-trials, with the latter requiring the inhibition of unwanted motor responses. In general, cognitive ERPs represent stimulus-locked time epochs in the EEG that can be related to distinct cognitive processes. (IV) In addition, a recent meta-analysis summarized previous study results on earlier versus later cognitive ERPs in ADHD compared to non-ADHD populations [32]. Results show that for early ERPs ADHD patients present shorter Go-P100-latencies when compared to non-ADHD. For later ERPs, individuals with ADHD showed smaller Cue-P300-amplitudes, longer Go-P300-latencies, smaller NoGo-P300-amplitudes, longer NoGo-P300-latencies, smaller contingent negative variation (CNV-) amplitudes, and smaller Pe-amplitudes. These robust empirical features found in the field of EEG research provide a reliable framework for testing validity of EEG data.

Differences in EEG data quality might exist between different groups assessed within a study due to developmental aspects or psychiatric symptoms. However, these contaminations possibly represent valid, characteristic information of those developmental and/or psychiatric populations with a substantial marker value. Those EEG data quality differences between study populations might subsequently have an impact on between-group differences in classical EEG- and ERP-analyses, and the biomarkers identified. Extensive efforts were made in previous studies to follow standards, control for artifacts, and include sufficient uncontaminated EEG signals for analyses also in clinical contexts: (a) Adequate designs and homogeneous participant groups were selected, and (b) different techniques and pre-processing methods ensured sufficient (largely) artifact-free EEG. Nevertheless, systematic group differences or remaining subtle signal distortions might still affect the analyzed data. Only a few studies so far have explicitly addressed and modelled systematic effects of EEG signal contaminations/data quality on results obtained in subsequent analyses of EEG/ERP data (e.g., on spectral power; e.g., [32,33]) to demonstrate and quantify such effects experimentally. However, these studies are urgently needed to explore the additional explanatory value of data quality besides developmental processes linked to brain maturation and ADHD symptoms, and to establish a link between the quality of assessed EEG data and results from planned EEG/ERP analyses.

Here, we present EEG data quality parameters from a recently conducted multicenter project assessing children, adolescents, and adults with ADHD, as well as non-ADHD children in school-age as control group to give insights into data quality, participant-related and methodological variables influencing data quality, as well as possible validation analyses to link data quality and replication of previous study results. Furthermore, we evaluated the additional influence of data quality on results obtained from spectral power analyses of resting EEG data beyond effects due to maturational processes and symptom severity. We suggest deriving data quality indices after pre-processing of the raw data by defining data quality as how much of the raw data assessed could actually be included in the final analyses. We go beyond the absolute number of acceptable segments after data pre-processing that was often taken as an index of data quality in previous work, and divide it by the total number of segments assessed to get an idea of how much data are useable for subsequent analyses (percentage of artifact-free segments). Within this study we assess how demographic, participant-related clinical (age, ADHD symptom dimensions, medication status), and methodological (type of measurement, pre-processing method, measurement duration) variables influence EEG data quality. We further conduct validation analyses to replicate robust landmark effects typically reported in the EEG literature. Additionally, we relate data quality to results obtained in spectral power analyses from resting EEG data exploring additional effects of data quality besides maturation and ADHD symptoms on results.

## 2. Materials and Methods

### 2.1. Participants

Pseudonymized data of children, adolescents, and adults (6–45 years) with ADHD for the present study were obtained from the ESCAlife project (ESCAschool, ESCAadol, and ESCAlate trials), a multicenter study including 14 sites (involving Bochum/Hamm, Bonn, Essen, Frankfurt, Göttingen, Homburg, Köln, Mainz, Mannheim, Marburg, Oldenburg, Rostock, Tübingen, and Würzburg). Details regarding the study protocol, each age-trial, and data acquisition have been published previously [34,35,36,37]. Within ESCApreschool (3–6 years), no EEG data were collected. All studies were previously registered by the German Trial Register (reference numbers: DRKS00008973, DRKS00008974, DRKS00008975, at: https://www.drks.de/drks_web/). Ethics approval was provided by the local ethical committees for each participating center, and written informed consent was obtained from the child, adolescent or adult. Furthermore, written assent was obtained from parents or guardians for participants below the age of 18 years. Exclusion criteria were: IQ < 80, diagnosis of pervasive developmental disorder, schizophrenia, bipolar disorder, severe depressive episode, epilepsy, heart disease, current or planned intensive behavioral therapy for ADHD or oppositional behavior on a weekly basis, for children with severe ADHD known non-response to all standard ADHD medication (methylphenidate, dexamphenidate, and atomoxetine), psychotropic medication (other than for ADHD) or neuroleptic medication (other than for the treatment of disturbance of impulse control), insufficient German language and reading skills of parents. IQ < 80, and insufficient German language skills were chosen as selection criteria as they were deemed relevant for participation in planned study-assessment (filling out questionnaires or understanding test instructions) and therapeutic interventions. EEG data within the ESCAlife-study were included from participants of the ESCAbrain-trial assessing the neurobiological underpinnings of ADHD, and the potential predictive value of neuronal markers for non-pharmacological treatment options. EEG data were assessed before (pre assessment) and after (post assessment) an intense, non-pharmacological intervention involving behavioral therapy (BT) or neurofeedback (NF) therapy. Finally, data from *n* = 184 ADHD children (age in years: M = 8.99, SD = 1.59), *n* = 39 adolescents with ADHD (age in years: M = 14.13, SD = 1.52), and *n* = 57 ADHD patients in adulthood (age in years: M = 29.39, SD = 6.73) were included in the current analyses. Furthermore, only at Mannheim center, 25 non-ADHD controls without any psychiatric disorder between the age of 6.00 and 11.11 years (non-ADHD controls) were assessed (age in years: M = 8.63, SD = 1.47), and EEG data were collected at two time points. Post assessments were done approximately 6 months after pre assessment. As the focus of the trials was on longitudinal aspects and changes due to different evidence-based ADHD interventions rather than on case-control comparisons, the study protocols did not include non-ADHD controls. Due to limited resources, non-ADHD controls could only be added for children, who form the largest and best studied age group regarding ADHD, although we acknowledge that a fully factorial design with controls in each age group would have been preferable.

### 2.2. Assessment of Demographic Information, and Clinical Characterization

Demographic information including age was assessed within an interview prior to any treatment or measurement. For clinical characterization and assessment of ADHD symptoms, several scales and interviews were used (see Appendix A).

### 2.3. EEG Data Acquisition

EEG data were acquired at each of the involved sites with NEUROPRAX or THERAPRAX full-band DC-EEG amplifier systems (with a high input impedance >10 GΩ for proprietary impedance control; neuroCare GmbH, Germany). Resting state data were collected with patients first having their eyes open and then eyes closed, four minutes each. The resting-state EEG was recorded using a 22-channel EEG cap (Brain Products, Gilching, Germany), and a sampling rate of 256 Hz (DC−70 Hz). A cued Continuous Performance Task (CPT) was used to probe preparatory and inhibitory neurophysiological activity (see Appendix A, for a detailed description). The EEG while performing the cued CPT (in ADHD children from ESCAschool/non-ADHD control children) or the Flanker-version (for adolescents and adults from ESCAadol, and ESCAlate, respectively) was recorded using a higher sampling rate of 512 Hz. Impedances were kept below 20 kΩ.

### 2.4. Data Preparation

EEG data were pre-processed using BrainVision Analyzer (Version 2.1) including the following pre-processing steps for the raw EEG signal: Offline filtering using Butterworth Zero Phase filters, a high-pass filter of 0.01 Hz (24 dB/oct), and a low-pass filter of 70 Hz (24 dB/oct). Furthermore, a notch filter of 50 Hz was applied. At first, data were inspected to reject the noisiest segments. Subsequently, for correction of ocular blinks and eye movements, an independent component analysis (ICA) was conducted based on a case-wise visual inspection. Then, data were re-referenced to the average, and segmented (division in equal sized components of 2.048 s for resting-state data). Further, an automatic artifact-detection method was applied using an exclusion criterion of ±150 µV. For later assessing the effects of different ocular correction methods, the same steps were repeated, but instead of using ICA decomposition, ocular blinks and eye movements were removed by the procedure described by Gratton and colleagues [38].

To assess data quality after implementing all steps of pre-processing, the number of good sweeps (< ±150 µV) was divided by the total number of sweeps assessed (percentage of artifact-free segments).

Regarding the validation analyses, for resting-state data, frequency band analyses using fast-Fourier transformation (FFT) were carried out, with data being divided into beta (12.5–30 Hz), alpha (7.5–12.5 Hz), theta (3.5–7.5 Hz), and delta (0.5–3.5 Hz) frequency bands, focusing on the Fz, Cz, and Pz electrode locations. For task-related data assessed using the cued CPT/Flanker-version of CPT, event-related potentials were extracted, focusing on the P300 component, as well as the CNV. For further analyses of time effects in the resting EEG eyes open and closed data, each data set was split in two time segments of equal size for the first half and second half of the measurement. Datasets for validation analyses were included with at least 20 segments per participant for resting data, and 10 segments for CPT data per condition, respectively.

For ERP-validation analyses, amplitudes and latencies were calculated for each participant for the Cue-P3 and the CNV components, as well as for Go- and NoGo-P3 components [30,39,40,41,42]. Cue-P3 peaks were identified at electrode Pz within a time window of 300–750 ms after cue onset. The CNV component was quantified at electrode Cz, and the most prominent statistical effects were expected within a time window of 1200–1650 ms after cue onset. Go-P3 and NoGo-P3 were defined as the most positive peaks at around 280–600 ms at electrode Pz and FCz, respectively. Amplitudes for all ERP components, and Cue-P3, Go-P3, and NoGo-P3 latencies were exported for further analysis.

### 2.5. Statistical Analyses

All statistical analyses were conducted using SPSS (version 24) and R software version 3.5.1.

To explore the effects of demographic and clinical variables on data quality, stepwise regression models were fitted to the data. To iteratively explore the influence of age, ADHD symptoms (inattention, hyperactivity/impulsivity; z-standardized to compare different scales used for children/adolescents, and adults, respectively), and medication status, those variables were sequentially entered into the model as predictor variables of interest. General linear models were used to analyze effects of condition, directly comparing eyes open versus eyes closed resting conditions, versus CPT. Furthermore, in paired-samples *t*-tests effects of different pre-processing methods, and measurement duration were explored. For exploring site effects on data quality within the current multicenter trial, again general linear models were fitted.

For validation analyses, correlational analyses (validation analysis I on age effects), paired-samples *t*-tests (validation analysis II on alpha blocking in transition from eyes closed to eyes open condition, and validation analysis III on CPT effects), as well as independent samples *t*-tests (validation analysis IV on ERP differences between ADHD and non-ADHD control children) were conducted. Validation analysis III and IV were conducted in children only. As *t*-tests were conducted for replication purposes or on different or only partly overlapping characteristics and datasets, no corrections for multiple testing were implemented.

To explore the additional effects of data quality on EEG power spectra besides demographic characteristics, stepwise regression models were used.

## 3. Results

### 3.1. Participant Characteristics

Demographic information on included participants can be found in Table 1.

### 3.2. Data Quality

#### 3.2.1. Descriptive Statistics

The first research question aimed at exploring data quality in children, adolescents, and adults with a diagnosis of ADHD, and school-age control children, and demographic, patient-related (age, medication, patient status), as well as methodological (type of measurement, pre-processing method, measurement duration) variables influencing data quality. Table 2 shows the descriptive statistics for each condition, and all (age) groups, respectively.

#### 3.2.2. Effects of Demographic and Clinical Information on Data Quality

A stepwise multiple regression was conducted to explore whether age, ADHD symptoms (inattention, hyperactivity/impulsivity), and medication status predict data quality. For pre eyes open, the regression model revealed at step 1, age contributed significantly to the regression model, F(1,224) = 5.41, *p* = 0.021), and accounted for 2.4% of the variation in data quality. Introducing ADHD symptoms explained an additional 6.1%, and this change in *R*^2^ was significant, F(3,222) = 6.84, *p* < 0.0001. This effect was primarily driven by adding hyperactivity/impulsivity to the regression model, t = −2.62, *p* < 0.01 (inattention: *p* > 0.05). Finally, the addition of medication explained an additional 0.6% of variation, but the change in *R*^2^ was not significant, *p* > 0.05.

For pre eyes closed, the regression model revealed at step 1, age contributed significantly to the regression model, F(1,223) = 15.52, *p* < 0.001, and accounted for 6.5% of the variation in data quality. Introducing ADHD symptoms explained an additional 7.1%, and this change in *R*^2^ was significant, F(3,221) = 11.58, *p* < 0.0001. This effect was primarily driven by adding hyperactivity/impulsivity to the regression model, t = −2.40, *p* = 0.017 (inattention: *p* > 0.05). Finally, the addition of medication explained an additional 2.0% of variation, and this change in *R*^2^ was again significant, F(4,220) = 10.18, *p* < 0.0001. When all independent variables were included at stage 3, a significant effect was revealed for symptoms of inattention additionally, t = −2.19, *p* = 0.03.

When exploring pre CPT data quality, at step 1 in the regression model, a significant effect of age was found, F(1,223) = 17.27, *p* < 0.001, accounting for 7.2% of variance. Adding ADHD symptoms explained further 7.1% with a significant change in *R*^2^, F(3,221) = 12.29. *p* < 0.001. Again, this effect was primarily driven by adding hyperactivity/impulsivity to the regression model, t = −2.60, *p* = 0.010 (inattention: *p* > 0.05). Finally, the addition of medication explained an additional 0.9% of variation, but the change in *R*^2^ was not significant, *p* > 0.05.

**Figure A1:**
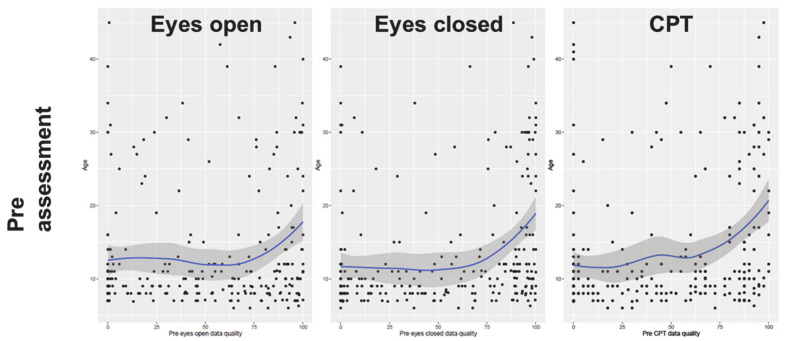
Association between age and data quality for all conditions at pre-assessment, respectively.

**Figure A2:**
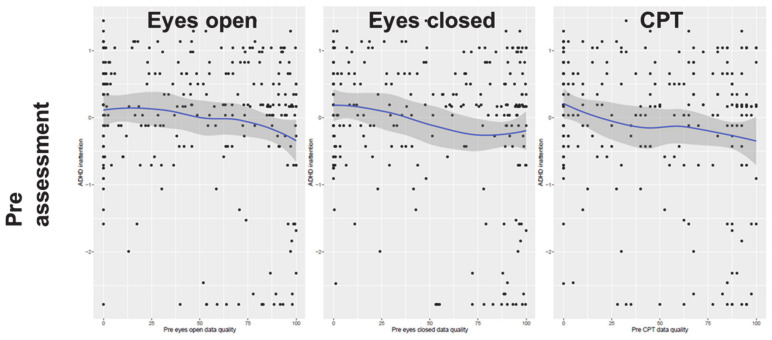
Association between ADHD symptoms of inattention and data quality for all conditions at pre-assessment, respectively.

**Figure A3:**
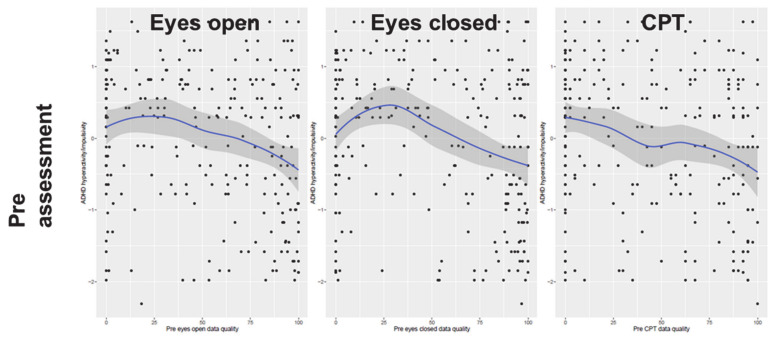
Association between ADHD symptoms of hyperactivity/impulsivity and data quality for all conditions at pre-assessment, respectively.

**Figure A4:**
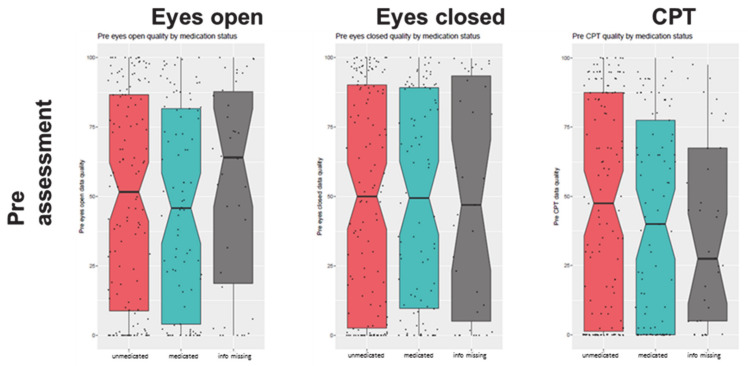
Differences in data quality for medication status at pre-assessment, respectively.

Appendix B (Figure A1, Figure A2, Figure A3 and Figure A4) shows the associations between demographic variables of interest and EEG data quality.

#### 3.2.3. Effects of Condition and Further Methodological Variables on Data Quality

For analyzing the effects of condition (directly comparing eyes open versus eyes closed versus CPT) across all participants, a general linear model was used. No significant effect was obtained, indicating no differences in data quality for different measurement conditions, *p* > 0.05.

Paired-samples *t*-tests were used to explore differences in data quality for different pre-processing methods (semiautomatic ICA versus automatic correction according to Gratton and Coles). Results show no significant differences for different ocular movement correction methods, *p* > 0.05.

To analyze differences in data quality for measurement duration, paired-samples *t*-tests were applied. No significant differences emerge for neither eyes open, nor eyes closed condition at pre assessment, *p* > 0.05.

Descriptive statistics and detailed results can be found in Appendix C (Table A1 and Table A2). Results for the effects of study-site on data quality are also presented in Appendix C.

### 3.3. Validation Analyses

#### 3.3.1. Validation Analysis I: Correlation between EEG Power Spectra and Age

**Figure 1:**
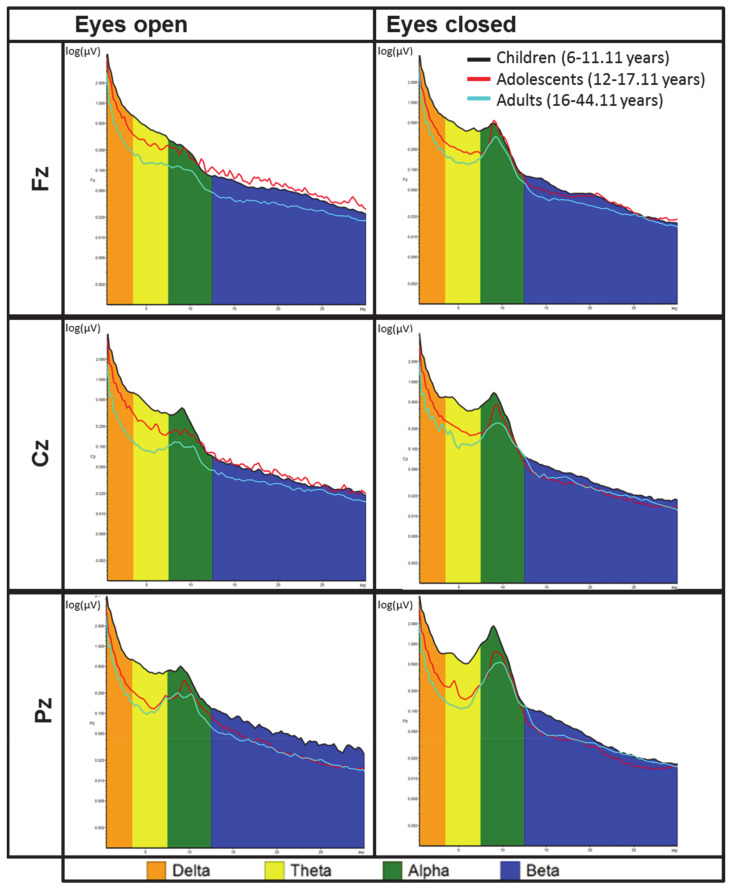
Averaged log10-transformed absolute spectral power in beta (12.5–30 Hz), alpha (7.5–12.5 Hz), theta (3.5–7.5 Hz), and delta (0.5–3.5 Hz) frequency bands for ADHD children (black), adolescents (red), and young adults (blue), respectively.

**Figure A5:**
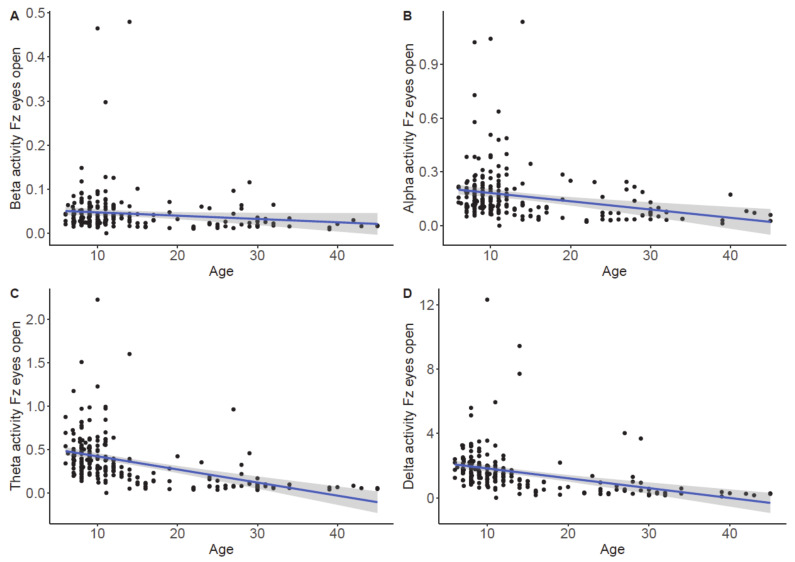
Association between age and spectral power in beta (**A**), alpha (**B**), theta (**C**), and delta (**D**) frequency bands for eyes open data at electrode position Fz.

**Figure A6:**
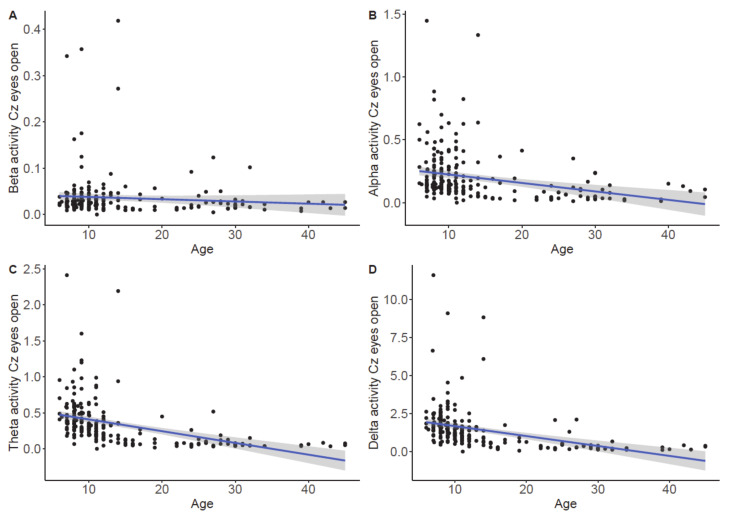
Association between age and spectral power in beta (**A**), alpha (**B**), theta (**C**), and delta (**D**) frequency bands for eyes open data at electrode position Cz.

**Figure A7:**
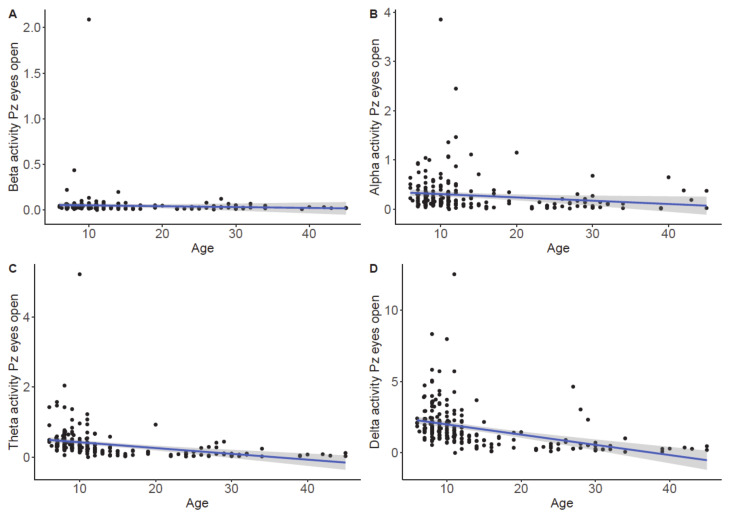
Association between age and spectral power in beta (**A**), alpha (**B**), theta (**C**), and delta (**D**) frequency bands for eyes open data at electrode position Pz.

**Figure A8:**
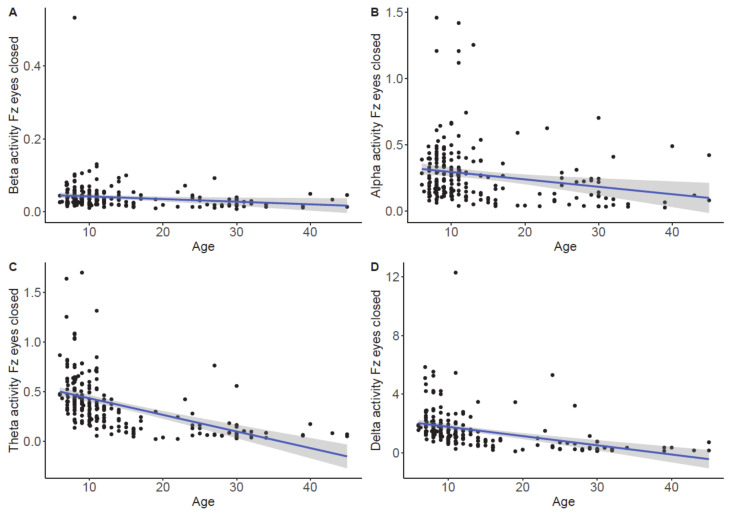
Association between age and spectral power in beta (**A**), alpha (**B**), theta (**C**), and delta (**D**) frequency bands for eyes closed data at electrode position Fz.

**Figure A9:**
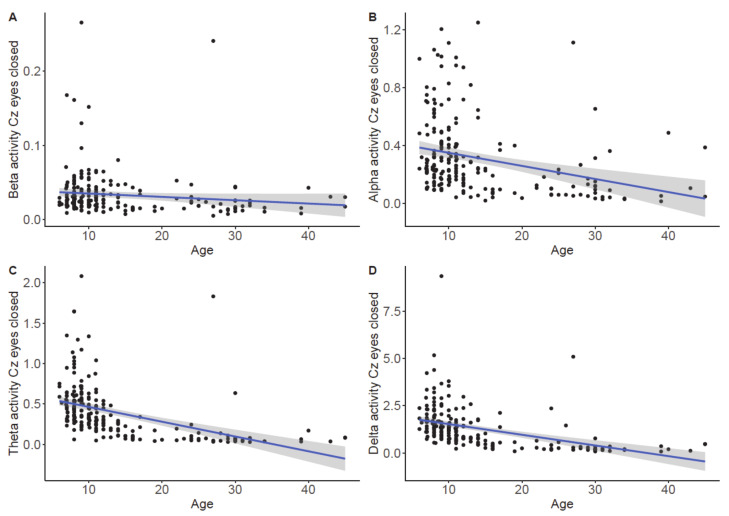
Association between age and spectral power in beta (**A**), alpha (**B**), theta (**C**), and delta (**D**) frequency bands for eyes closed data at electrode position Cz.

**Figure A10:**
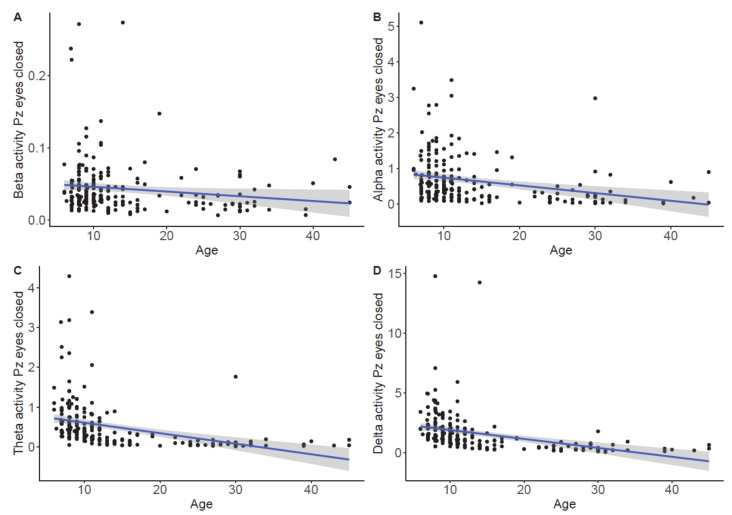
Association between age and spectral power in beta (**A**), alpha (**B**), theta (**C**), and delta (**D**) frequency bands for eyes closed data at electrode position Pz.

**Figure A11:**
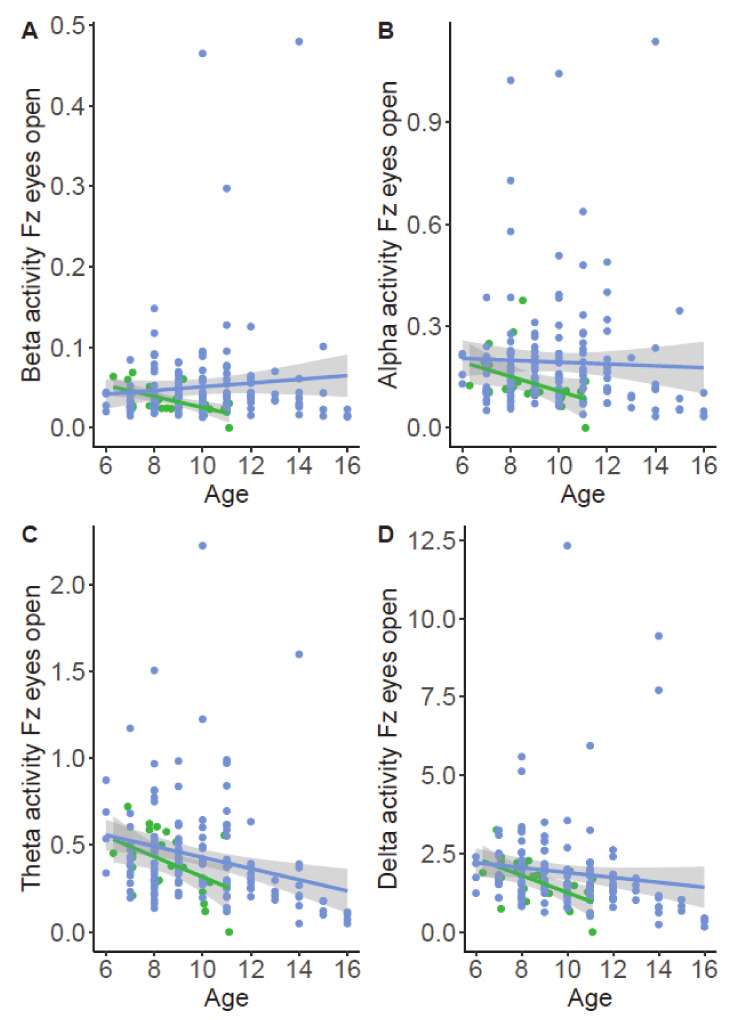
Association between age and spectral power in beta (**A**), alpha (**B**), theta (**C**), and delta (**D**) frequency bands for eyes open data at electrode position Fz for ADHD (blue) and non-ADHD (green) children and adolescents <16 years of age.

**Figure A12:**
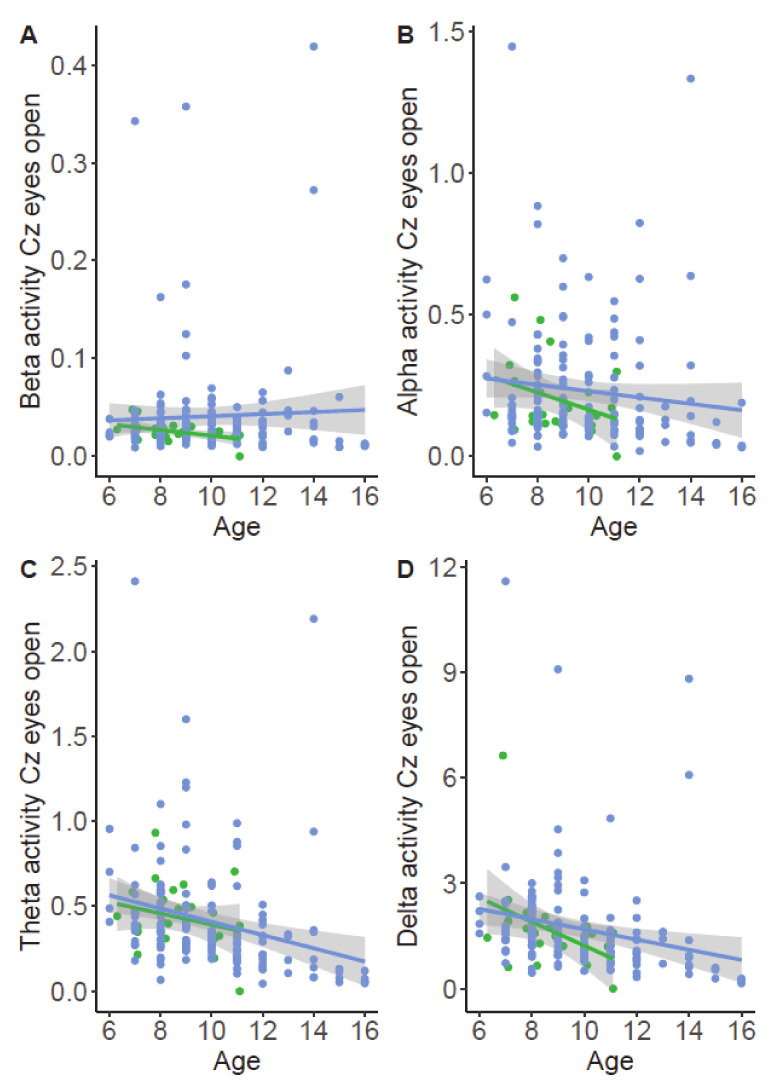
Association between age and spectral power in beta (**A**), alpha (**B**), theta (**C**), and delta (**D**) frequency bands for eyes open data at electrode position Cz for ADHD (blue) and non-ADHD (green) children and adolescents <16 years of age.

**Figure A13:**
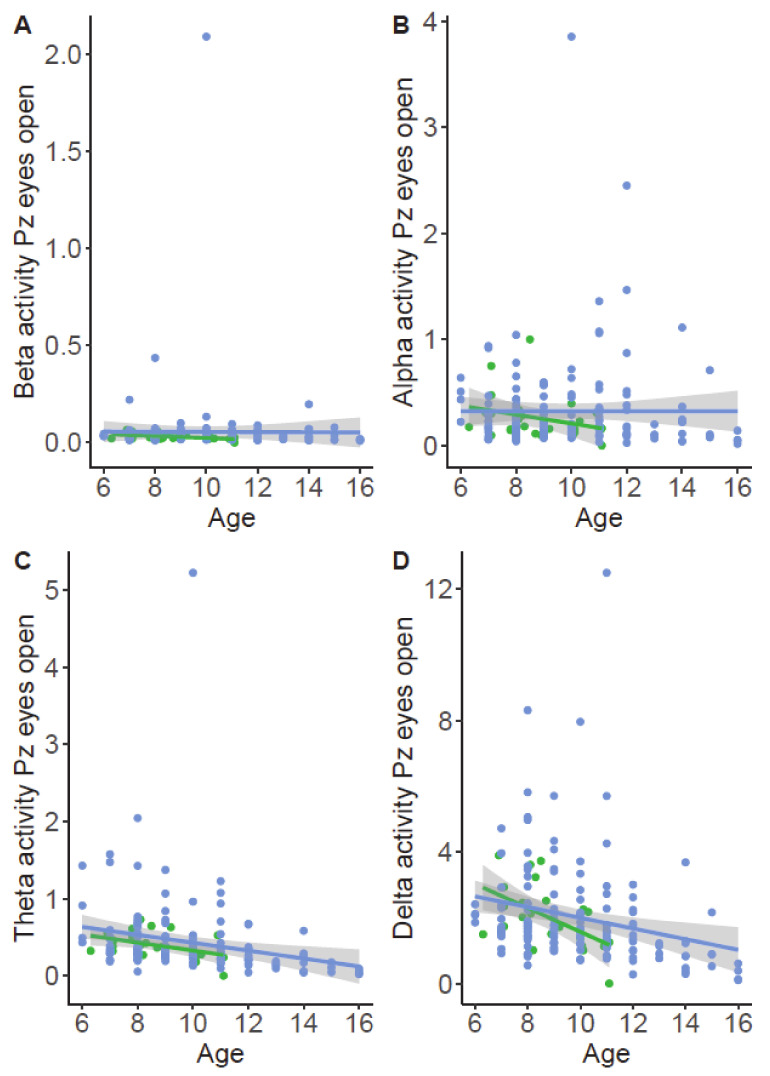
Association between age and spectral power in beta (**A**), alpha (**B**), theta (**C**), and delta (**D**) frequency bands for eyes open data at electrode position Pz for ADHD (blue) and non-ADHD (green) children and adolescents <16 years of age.

**Figure A14:**
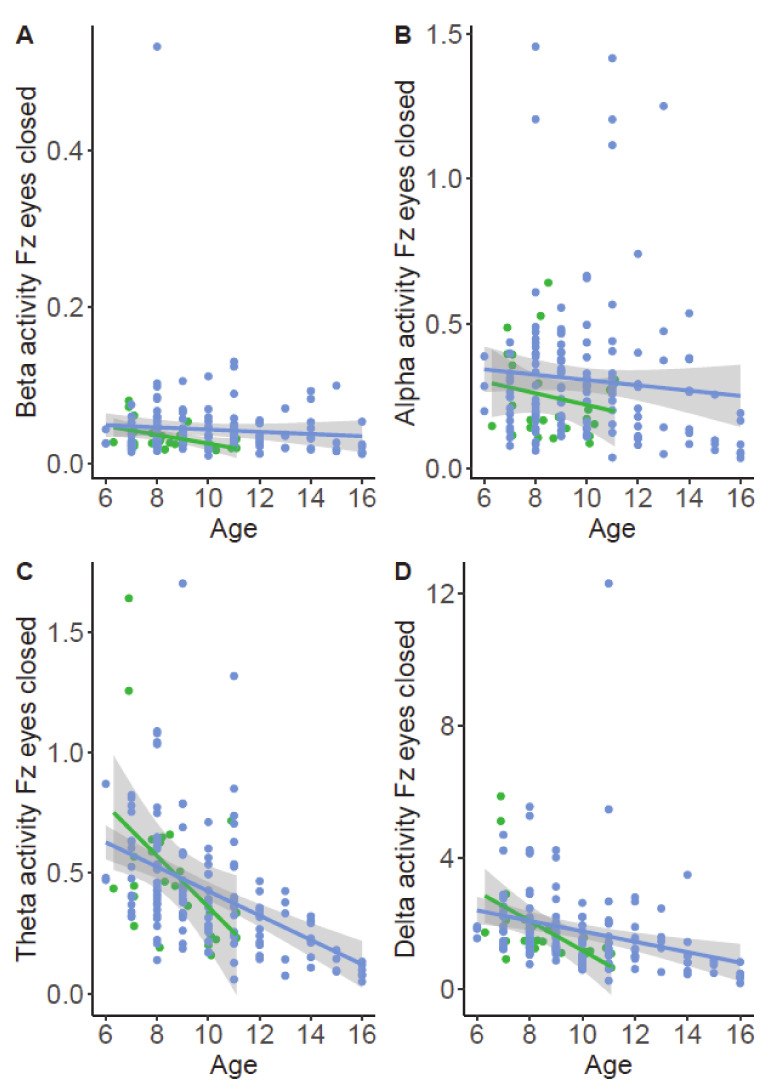
Association between age and spectral power in beta (**A**), alpha (**B**), theta (**C**), and delta (**D**) frequency bands for eyes closed data at electrode position Fz for ADHD (blue) and non-ADHD (green) children and adolescents <16 years of age.

**Figure A15:**
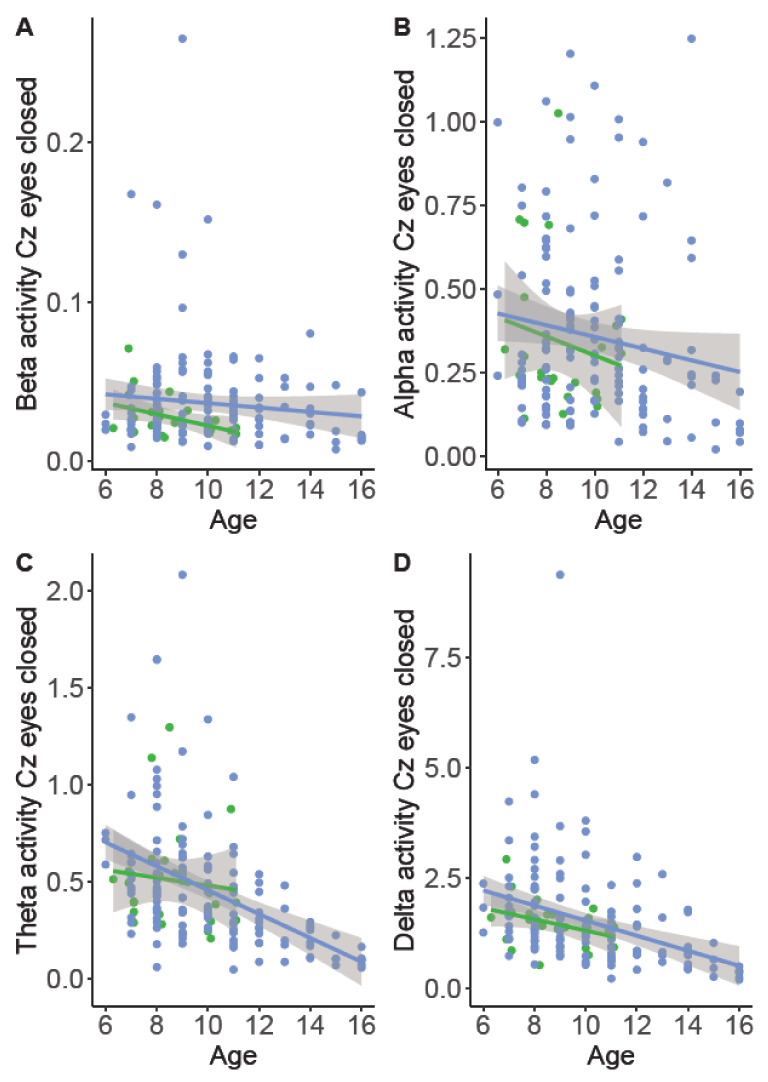
Association between age and spectral power in beta (**A**), alpha (**B**), theta (**C**), and delta (**D**) frequency bands for eyes closed data at electrode position Cz for ADHD (blue) and non-ADHD (green) children and adolescents <16 years of age.

**Figure A16:**
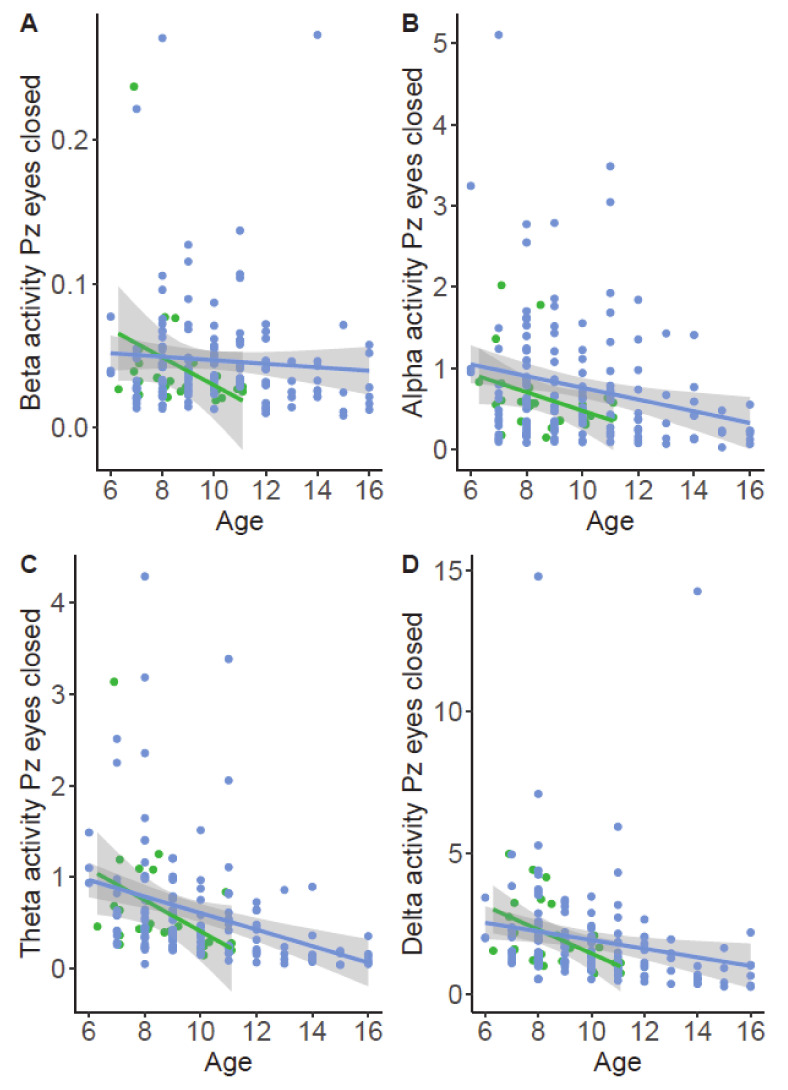
Association between age and spectral power in beta (**A**), alpha (**B**), theta (**C**), and delta (**D**) frequency bands for eyes closed data at electrode position Pz for ADHD (blue) and non-ADHD (green) children and adolescents <16 years of age.

Significant small to moderate negative correlations were obtained between age and eyes open alpha activity, eyes open theta activity, and eyes open delta activity at Fz, Cz, and Pz at pre assessment. For eyes closed, significant negative correlations were obtained between age and alpha activity, theta activity, and delta activity at Fz, Cz, and Pz, respectively. In addition, small negative correlations at Fz, and Pz electrode positions were identified for beta activity. These results indicate that with increasing age, power in alpha, theta, and delta bands decreases. Results across all participants are shown in Table 3. Figure 1 presents absolute spectral power (log-transformed values are displayed for illustrative purposes) in all frequency bands of interest for ADHD children, adolescents, and adults, respectively. Appendix D (Figure A5, Figure A6, Figure A7, Figure A8, Figure A9 and Figure A10; Table A3 and Table A4) shows the associations between age and EEG spectral power in resting conditions across all participants. Further, for the purpose of comparison with previous literature [23], Appendix D (Figure A11, Figure A12, Figure A13, Figure A14, Figure A15 and Figure A16) presents results from correlational analyses with only children and adolescents included (<16 years of age), separately for ADHD groups and the non-ADHD control children, as typically substantially higher associations are identified for younger age groups and in non-ADHD control groups.

#### 3.3.2. Validation Analysis II: Alpha Blocking in Transition from Eyes Closed to Eyes Open Condition (Alpha Reactivity)

**Figure 2:**
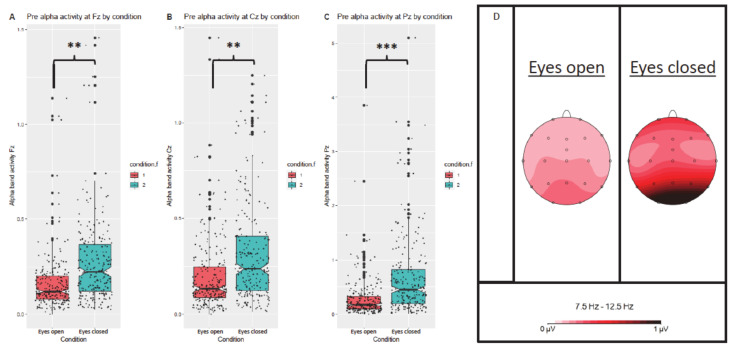
Differences in alpha activity by condition at Fz (**A**), Cz (**B**), and Pz (**C**) electrode locations. Corresponding topographical maps in the alpha frequency range of 7.5–12.5 Hz for eyes open, and eyes closed conditions, respectively (**D**). *** *p* ≤ 0.0001, ** *p* ≤ 0.001, ° *p* ≤ 0.05.

Paired samples *t*-Tests were conducted to compare FFT alpha activity in eyes open versus eyes closed conditions at electrode locations Fz, Cz, and Pz, respectively. There was a significant difference in alpha activity at all three electrode positions for eyes open (Fz: M = 0.15, SD = 0.13; Cz: M = 0.19, SD = 0.19; Pz: M = 0.25, SD = 0.26) and eyes closed (Fz: M = 0.27, SD = 0.22; Cz: M = 0.32, SD = 0.27; Pz: M = 0.68, SD = 0.75), t(208) = −8.549, *p* < 0.001 at Fz, t(208) = −9.168, *p* < 0.001 at Cz, and t(208) = −9.783, *p* < 0.0001 at Pz, respectively. These results indicate an increase in alpha activity at all three electrode locations from eyes open to eyes closed condition (see also Figure 2).

Furthermore, a decrease in frontal beta, an increase in posterior beta, and an increase in central and posterior theta activity from eyes open (M = 0.04, SD = 0.04, M = 0.03, SD = 0.03, M = 0.33, SD = 0.30, M = 0.32, SD = 0.26) to eyes closed condition (M = 0.04, SD = 0.02, M = 0.04, SD = 0.03, M = 0.38, SD = 0.31, M = 0.48, SD = 0.52) was obtained, t(208) = 2.281, *p* = 0.024, t(208) = −4.203, *p* < 0.001, t(208) = −2.784, *p* = 0.006, t(208) = −5.832, *p* < 0.001, respectively.

#### 3.3.3. Validation Analysis III: CPT Task Effect: Comparison between Go- and Nogo-P3 Amplitude at Pz

**Figure 3:**
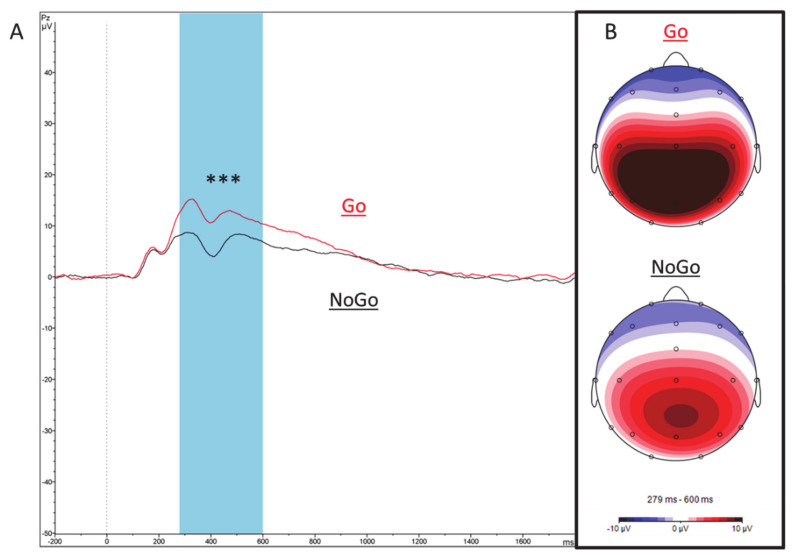
ERP Go- and NoGo-P3 components in children. (**A**) Stimulus-locked ERP wave shapes of the Go- (red) and NoGo-P3 (black) components at electrode Pz. (**B**) Corresponding maps in the time range of the Go- and NoGo-P3 (280–600 ms). *** *p* ≤ 0.0001, ° *p* ≤ 0.05.

Paired-samples *t*-tests were used to explore mean amplitude differences at Pz electrode for Go- and NoGo-P3. Results show a significant difference between Go- and NoGo-P3 mean activity at posterior regions, with a substantially higher Go-P3 mean amplitude (M = 20.00, SD = 6.65) compared to the NoGo-P3 component (M = 14.64, SD = 7.27), t(128) = 9.402, *p* < 0.0001, see Figure 3.

#### 3.3.4. Validation Analysis IV: ERP Differences between Children with ADHD and Non-ADHD Controls

Descriptive statistics for ERP amplitudes and latencies of interest can be found in Table 4.

Peak definition: Cue-P3 at Pz within a time window of 300–750 ms after cue onset. CNV at Cz, within a time window of 1200–1650 ms after cue onset. Go-P3 and NoGo-P3 at around 280–600 ms at electrode Pz and FCz, respectively.

**Figure 4:**
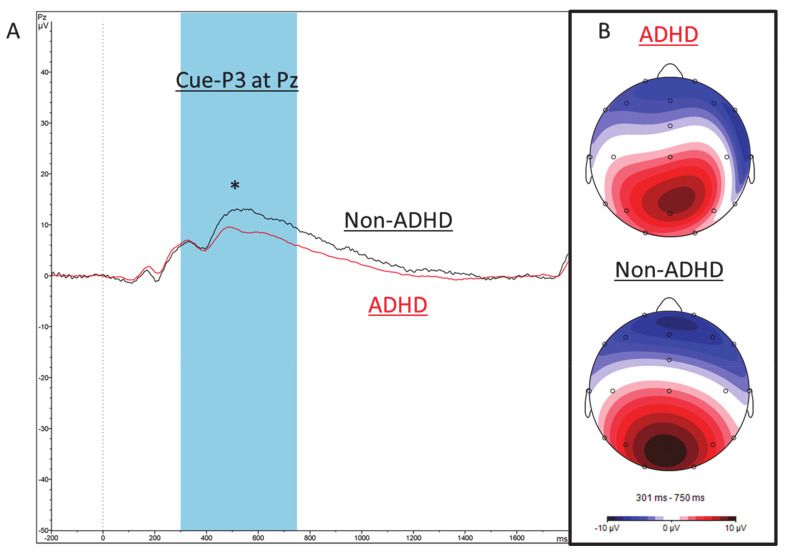
ERP Cue-P3 component for ADHD and non-ADHD control children. (**A**) Stimulus-locked ERP wave shapes of the Cue-P3 component for ADHD patients (red), and non-ADHD control children (n = 24; black) at electrode Pz. (**B**) Corresponding maps in the time range of the Cue-P3 (300–750 ms). * *p* ≤ 0.01, ° *p* ≤ 0.05.

Comparing children with ADHD to non-ADHD control children in school-age, a significant between-group difference was obtained for the Cue-P3 amplitude, t(145) = 2.37, *p* = 0.019, indicating smaller Cue P3-amplitudes in ADHD children (M = 13.31, SD = 5.43) compared to non-ADHD ESCAschool-controls (M = 16.11, SD = 5.04; see Figure 4). No further differences were obtained for other ERP components.

### 3.4. The Additional Influence of Data Quality on Power Spectra (FFT) Results in Eyes Open and Eyes Closed Resting Conditions

A stepwise multiple regression was conducted to explore whether age, ADHD symptoms (inattention, hyperactivity/impulsivity), and data quality predict FFT power spectra from resting-state measurements.

For pre eyes open data quality, the models revealed an additional significant effect of data quality in step 3 for alpha activity, as well as for beta activity at Fz, and Cz, respectively (alpha: Δ*R*^2^ = 0.043, *p* = 0.001, and Δ*R*^2^ = 0.027, *p* = 0.011; and beta: Δ*R*^2^ = 0.035, *p* = 0.005, and Δ*R*^2^ = 0.080, *p* < 0.0001, respectively). In addition, significant effects were obtained for theta, and delta frequency bands at electrode positions Fz, Cz, and Pz (theta: Δ*R*^2^ = 0.035, *p* = 0.001, Δ*R*^2^ = 0.029, *p* = 0.005, and Δ*R*^2^ = 0.016, *p* = 0.047; delta: Δ*R*^2^ = 0.051, *p* < 0.0001, Δ*R*^2^ = 0.059, *p* < 0.0001, and Δ*R*^2^ = 0.063, *p* < 0.0001, respectively). Furthermore, for pre eyes closed data quality, a significant additional predictive value was obtained for beta, theta, and delta activity at electrode positions Fz, Cz, and Pz (beta: Δ*R*^2^ = 0.026, *p* = 0.019; Δ*R*^2^ = 0.074, *p* < 0.0001; Δ*R*^2^ = 0.081, *p* < 0.001; theta: Δ*R*^2^ = 0.052, *p* < 0.001; Δ*R*^2^ = 0.049, *p* < 0.0001; Δ*R*^2^ = 0.039, *p* = 0.002; and delta: Δ*R*^2^ = 0.082, *p* < 0.001; Δ*R*^2^ = 0.078, *p* < 0.0001; Δ*R*^2^ = 0.082, *p* < 0.001). Appendix E (Table A5, Table A6, Table A7, Table A8, Table A9, Table A10, Table A11, Table A12, Table A13, Table A14, Table A15, Table A16, Table A17, Table A18, Table A19, Table A20, Table A21, Table A22, Table A23, Table A24, Table A25, Table A26, Table A27 and Table A28) shows full details of the results obtained from the stepwise multiple regression models.

To explore the association of data quality and spectral power in more detail, post-hoc correlational analyses were conducted. As revealed by those analyses, data quality is negatively correlated with spectral power for all significant results across all different bands for both conditions, indicating that lower data quality is associated with higher spectral power.

## 4. Discussion

### 4.1. Summary of Results and Interpretation

The first aim of this study was to explore EEG data quality parameters in a multicenter study of children, adolescents, and adults with ADHD, and a non-ADHD school-age control sample, and to analyse the potential influence of participant-related and methodological variables. Data quality was defined as the percentage of artifact-free segments in the EEG after pre-processing. The current study found that across assessments, and most of the measurement conditions, the percentage of artifact-free segments was related to age, and symptoms of hyperactivity/impulsivity. Age is positively associated with data quality, indicating higher data quality with increasing age. For symptoms of hyperactivity/impulsivity, a negative association was obtained, pointing out that with increasing symptoms of hyperactivity/impulsivity the percentage of artifact-free segments decreases. For eyes open data, the association between EEG data quality and ADHD symptoms of hyperactivity/impulsivity was even stronger than for age, whereas for the eyes closed and CPT conditions effects were comparable for those participant-related characteristics. This might possibly be due to sequence effects, with increasing time since the start of the first measurement (resting with eyes open), developmental effects becoming more relevant. Further, for eyes closed data quality, symptoms of inattention seem to play an additional role, with higher symptoms being related to lower data quality. A possible explanation might be that attentional processes are more involved in successfully accomplishing the task of resting with eyes holding closed (e.g., not to move, not to fall asleep). In addition, it is important to note that there are substantial age effects across all task conditions that do not differ between rest conditions and the CPT, even though a more demanding Flanker-version of the CPT was used for adolescents and adults. Rather than representing a challenge due to task-inherent demands, the 11 min of task completion for the CPT might have caused boredom in children contributing to the reported effect. No significant effect was obtained for condition or any of the methodological influence variables of interest explored within the current trial. No significant data quality differences were obtained for the direct comparison of the three conditions (eyes open versus eyes closed versus CPT, always applied in the same order) across all participants assessed. This indicates that neither task demands nor time effects seem to have a substantial impact on data quality across all participants. From these results it can be suggested that whereas participant-related characteristics have a strong impact on data quality, the methodological differences regarding study design explored here play a minor role for reliability of EEG study results.

A further objective of this study was to replicate landmark effects typically reported in the EEG literature to prove validity of data. Effects from maturational processes, task demands, and ADHD status have been explored. In line with previous findings, the results of these analyses show that age is negatively associated with EEG spectral power: With increasing age EEG power decreases, especially for slow oscillatory activity activity (theta and delta bands). However, correlations found here are a bit lower than reported previously [24]. This is probably due to a different age range of the assessed ADHD-study population, and symptoms of ADHD with ADHD-patients typically showing lower associations. Furthermore, comparing the alpha reactivity between eyes open and eyes closed conditions, we found an increase in alpha activity in the transition from eyes open to eyes closed replicating previous robust findings on the alpha blocking phenomenon. Additionally, validity analyses addressing robust CPT-effects showed a significantly higher Go-P3 amplitude compared to the NoGo-P3 at posterior regions replicating previous findings on task manipulation effects. Finally, in line with a recent meta-analysis [32] we found a significant difference in the Cue-P3 amplitude component between children with ADHD and non-ADHD controls, with higher amplitudes in control participants. However, no significant differences were obtained for other ERP components possibly due to different developmental effects. By replicating those landmark effects, we can infer substantial validity of current data allowing for subsequent analyses and valid interpretations, and further, established a link between data quality and replication of previous study results.

Finally, this study aimed to determine the additional effects of data quality on FFT spectral power beyond maturational effects and effects due to symptom severity. As indicated by the stepwise regression models, data quality has a relevant additional impact on spectral power for eyes open, and eyes closed data. As shown by post-hoc correlational analyses, the associations between data quality and FFT spectral power are negative indicating higher activity in frequency band power with lower data quality. For alpha and beta frequency bands in eyes open datasets, this result might be explained by the fact that those bands include the highest frequency band widths ranging from 7.5 to 12.5 Hz and 12.5 to 30 Hz, respectively. These higher frequency band ranges might be more affected by myogenic activity near the head with a high-frequency activity of >20 Hz [33]. This increased myogenic activity might consequently lead to a lower percentage of artifact-free segments influencing results obtained in FFT analyses, such as diluting or mimicking EEG alpha or beta rhythms.

### 4.2. Relevance of Results and Practical Implications

The findings of the current study highlight the relevance of explicit data quality assessments in EEG studies, especially when younger populations are in the focus of interest, and when psychiatric samples are explored prone to EEG artifacts. It is interesting to note, that while participant-related characteristics have a substantial impact on data quality, reliability, and consequently the interpretability of findings, the methodological variables explored here have not. This finding has a highly important impact on the process of study implementation including the planning of data pre-processing strategies. It seems especially relevant that demographic and clinical characteristics of participant samples included in studies are reported explicitly in publications: Effects can be classified more accurately, and addressed in replication studies as well as in reviews and meta-analytic approaches. Nevertheless, future studies should assess further different methodological variables, and efforts on methodological standardization for a higher comparability of study results should moreover be strengthened [43,44].

By replicating robust landmark effects of the EEG literature, we were able to prove validity of current datasets, and to ensure valid conclusions drawn from subsequent analyses. Ensuring reliability and validity of assessed data has substantial implications for the status quo of a research field. They allow for valid interpretation of study results, and a higher application value, e.g., for deep-learning approaches [45]. This finding further highlights that large-scale multicenter studies on ADHD patients prone to EEG artifacts are feasible. This feasibility is urgently needed for further detailed explorations of the diagnostic and predictive value of EEG/ERP markers for this highly prevalent neurodevelopmental disorder.

The finding of an additional effect of our data quality index on FFT spectral power beyond maturational processes and symptoms of ADHD points out to the need for discussing and challenging EEG results on spectral power as dependent variable, especially for classification purpose. This result might be due to myogenic activity as a potential confounder (diluting or mimicking spectral power effects) contaminating the EEG signal. Nevertheless, those indices might be of value for characterizing psychiatric patients, especially, when motor activity represents a central characteristic of clinical populations explored. They might be of additional value for classification purposes and for differentiating clinical from non-clinical groups, as well as between different clinical groups. In addition, controlling for EEG data quality seems to be urgently needed when spectral power analyses are conducted.

### 4.3. Limitations and Future Directions

A few limitations of the current work have to be mentioned. First, in our ADHD sample age was restricted from 6 to 45 years. No older adults were included, and only a few datasets for adolescents. Therefore, effects are primarily driven by data from children and young adults. This has to be taken into account when interpreting current results. Future studies are needed including a sample with a larger age range of included ADHD patients. Furthermore, only a small non-ADHD sample in school-age was recruited. Therefore, as we not have a full-factorial design of ADHD status across all age groups, patient-control comparisons in our validation analyses could only be conducted on children between 6 and 12 years of age. In future work, larger non-ADHD samples should be included spanning a broader age range.

Within the current study, the focus was set on a few potentially relevant participant-related and methodological variables influencing data quality. Besides those variables addressed within the current work, others might be relevant. Further studies are needed at this stage. In addition, the percentage of artifact-free segments was defined as the relevant index of data quality. There exist many more data quality indices and further replication of current results is needed comparing different data quality indices. Within our analyses, no corrections for multiple testing were applied. However, as our analyses involved replications, and only partial overlap regarding characteristics and datasets within separate tests, this is not necessarily recommended.

Additionally, further EEG measures besides spectral power and ERP amplitudes and latencies might be of relevance for future work on data quality. For example, functional connectivity measures between different electrode locations could be assessed and analyzed in multivariate models in future studies as they might be relevant for a further characterization of ADHD. As we are explicitly interested in data quality effects, the current work focused on peak amplitudes rather than mean amplitudes as peak amplitudes are typically most affected by noise [46,47,48,49,50]. However, future work will also assess other ERP indices. In particular, besides peak amplitude data mean amplitudes of relevant ERP indices will be taken into account for a more robust and unbiased approach.

## 5. Conclusions

The current study contributes to our understanding of EEG data quality, participant-related and methodological variables influencing EEG data quality, and the additional effects of data quality on results obtained from FFT analyses beyond demographic and clinical characteristics. To the best of our knowledge, this is the first study explicitly investigating the impact of several study-specific variables on data quality in a large ADHD sample from 6 to 45 years of age. The results of this investigation show that on the one hand demographic variables, especially, age and symptoms of hyperactivity/impulsivity, have a substantial impact on data quality. On the other hand, methodological differences regarding study-design and analytical methods assessed here have not. Furthermore, the current work highlights the importance of replication analyses to prove validity of the assessed data. Additionally, we found that data quality substantially affects spectral power beyond patient-related characteristics pointing out to the need for cautious interpretations of results obtained in EEG analyses on frequency band power. These findings have a high relevance for the implementation of studies, analyzing and publishing EEG data, and for interpreting scientific results obtained from EEG studies. Further, current results show that with a careful design and systematic data quality control, informative large-scale multicenter trials on neurophysiological mechanisms in neurodevelopmental disorders across the lifespan are actually feasible. Nevertheless, results are restricted to the limitations discussed. Future studies are needed to replicate and extend current findings.

## Figures and Tables

**Table 1:** Demographic information.

	N	Age, M (SD)	ADHD Symptoms Inattention, M (SD) Hyperactivity/Impulsivity, M (SD)	Medication (%)
**Non-** **attention-deficit/hyperactivity disorder (ADHD) control children**	25	8.63 (1.47)	0.20 (0.28) 0.24 (0.23)	0 (0%)
**ADHD children from ESCAschool**	184	8.99 (1.59)	2.19 (0.40) 1.88 (0.70)	82 (55.78%)
**ADHD adolescents from ESCAadol**	39	14.13 (1.52)	2.05 (0.39) 1.42 (0.71)	15 (46.88%)
**ADHD adults from ESCAlate**	57	29.39(6.73)	7.81 (1.14) 5.28 (2.29)	8 (14.81%)

ADHD symptom scale ranges for non-ADHD controls, ADHD children from ESCAschool, and ADHD adolescents from ESCAadol: (0–3), for ADHD adults from ESCAlate: (0–10).

**Table 2:** Descriptive statistics for data quality index (percentage of artifact-free segments).

Pre Assessment			
	Eyes Open, M% (SD)	Eyes Closed, M% (SD)	CPT, M% (SD)
**Non-ADHD control children**	73.35% (27.33)	75.80% (26.49)	69.92% (26.79)
**ADHD children from ESCAschool**	41.28% (35.14)	37.27% (36.40)	30.96% (33.19)
**ADHD adolescents from ESCAadol**	54.84% (38.84)	58.12% (38.29)	49.61% (38.81)
**ADHD adults from ESCAlate**	57.78% (38.15)	66.61% (38.32)	64.63% (36.42)
**Total**	48.92% (36.87)	49.04% (38.95)	43.75% (37.42)

The pre assessment was carried out before the intense treatment schedule of the stepped-care treatment program within the ECSAlife study.

**Table 3:** Correlations between age and fast-Fourier transformation (FFT) frequency band activity at pre assessment.

	Eyes Open			Eyes Closed		
Electrode Location	Fz	Cz	Pz	Fz	Cz	Pz
**beta (μV) × age (years)**	−0.130	−0.088	−0.058	−0.154 °	−0.125	−0.151 °
**alpha (μV) × age (years)**	−0.265 ***	−0.297 ***	−0.155 °	−0.210 **	−0.299 ***	−0.265 ***
**theta (μV) × age (years)**	−0.461 ***	−0.452 ***	−0.324 **	−0.521 ***	−0.471 ***	−0.376 ***
**delta (μV) × age (years]**	−0.387 ***	−0.406 ***	−0.415 ***	−0.404 ***	−0.449 ***	−0.377 ***

Frequency band widths: beta (12.5–30 Hz), alpha (7.5–12.5 Hz), theta (3.5–7.5 Hz), and delta (0.5–3.5 Hz). Pearson product-moment correlations are displayed. *** *p* ≤ 0.0001, ** *p* ≤ 0.001, ° *p* ≤ 0.05.

**Table 4:** Event-related potential (ERP) amplitudes and latencies in ADHD (ESCAschool) and non-ADHD control children.

	ADHD Children			Non-ADHD Control Children			Comparison	
	N	M	SD	N	M	SD	t	*p*
**Contingent negative variation (CNV) amplitude**	122	−2.43 µV	4.59 µV	25	−3.07 µV	4.00 µV	−0.548	0.585
**Cue P3 amplitude**	122	13.31 µV	5.43 µV	25	16.11 µV	5.04 µV	2.503	0.013
**Cue P3 latency**	122	506.96	118.76 ms	25	531.09 ms	108.26 ms	0.728	0.468
**Go P3 amplitude**	105	19.79 µV	6.26 µV	25	20.92 µV	7.99 µV	1.006	0.316
**Go P3 latency**	105	390.12 ms	99.23 ms	25	420.63 ms	102.01 ms	1.024	0.308
**NoGo P3 amplitude**	109	11.07 µV	8.12 µV	25	11.81 µV	6.76 µV	0.406	0.686
**NoGo P3 latency**	109	441.10 ms	76.13 ms	25	449.61 ms	69.39 ms	0.608	0.544

## Data Availability

Due to ethical, legal or privacy issues are present, data should not be shared.

## Appendix A

### Appendix A.1. Assessment of Demographic Information, and Clinical Characterization

Depending on age group and subtrial, the following assessment methods were implemented: for ESCAschool (6;0–11;11 years of age): the clinical interview “Diagnose-Checkliste für Aufmerksamkeitsdefizit−/Hyperaktivitätsstörungen” (DCL-ADHS; Döpfner & Görtz-Dorten, 2017), the “Clinical Global Impression Scale–Severity” (CGI-S; National Institute of Mental Health, 1976), FBB-ADHS-Parent/-Teacher, FBB-SSV-Parent/-Teacher, SDQ-Parent; for ESCAadol (12;0–17;11 years of age): DCL-ADHS-clinical interview, CGI-S, SBB-ADHS, FBB-ADHS-Parent/-Teacher, SBB-SSV, FBB-SSV-Parent/-Teacher, SDQ-Parent; and for ESCAlate (16;0–44;11 years of age): ADHD-DCQ, IDA interview.

### Appendix A.2. EEG Data Acquisition—The Continuous Performance (CPT) Task

The cued Continuous Performance Task (CPT-OX/Flanker CPT-OX) was used to probe attention, preparation, and inhibitory activity at pre- (T2) and post-assessment (T3). In ESCAschool, the simple version of the cued CPT was used. For participants in the ESCAadol, and ESCAlate trials, the Flanker-version of the cued CPT was implemented. The task consists of 400 black letters or letter arrays (for the Flanker-version), made up of a central black letter (and for the Flanker version: Plus additional incompatible flankers on each side to increase difficulty). The presented letters or arrays include the cue letter ‘O’, the target letter ‘X’ as well as further distractors (‘H’, ‘B’, ‘C’, ‘D’, ‘E’, ‘F’, ‘G’, ‘J’, and ‘L’). For the Flanker version of the task in the ESCAadol, and ESCAlate trials, the cue and target letters (‘O’ and ‘X’, respectively) were flanked by distractor letters (‘XOX’ and ‘OXO’, respectively). Letters were presented every 1.650 ms for 150 ms in a pseudo-randomized order. Participants were instructed to respond as quickly as possible to cue-target sequences (‘O’–‘X’). 80 cues were followed by the target in 40 trials (Go condition), and by neutral distractors in the other 40 trials (NoGo condition). One minute of practice trials was implemented before the main task and repeated, if required, to ensure participant understanding of the task. Participants were instructed to respond to Cue-Go trials by pressing a button as quickly as possible with the index finger of their preferred hand. Task duration was approximately 11 min.

## Appendix C

### Appendix C.1. Data Quality—Effects of Methodological Variables

#### Appendix C.1.1. Effects of Pre-Processing/Ocular Correction Method

**Table A1:** Descriptive statistics: Effects of pre-processing/Ocular correction method (ICA versus Gratton and Coles)—non-ADHD controls only.

	Ocular Correction Method	N	Data Quality, M (%)	Data Quality, SD (%)
**Eyes open**	Gratton and Coles	24	75.85%	25.42%
	ICA	24	73.37%	27.32%
**Eyes closed**	Gratton and Coles	25	76.52%	25.54%
	ICA	25	75.80%	26.49%

#### Appendix C.1.2. Effects of Measurement Duration

**Table A2:** Descriptive statistics: Effects of measurement duration (segment 1 versus segment 2).

	Segment	N	Data Quality, M (Absolute Number)	Data Quality, SD (Absolute Number)
**Eyes open**	**Segment 1**	251	75.55	54.19
	**Segment 2**	222	80.43	55.75
**Eyes closed**	**Segment 1**	247	76.49	56.13
	**Segment 2**	216	79.85	58.26

#### Appendix C.1.3. Effects of Site

General linear models were used to explore effects of site on data quality for eyes open, eyes closed, and the CPT task-condition, respectively. Significant effects were identified for all conditions: Eyes open, F(15,251) = 3.2219, *p* < 0.0001, eyes closed, F(15,247) = 4.2029, *p* < 0.0001, and the CPT, F(13,251) = 4.9926, *p* < 0.0001. However, as the study sites did not include participants for all age trials, site represents a confounded variable, and was therefore not included in subsequent analyses.

## Appendix D

### Associations between Age and EEG Spectral Power in Resting Conditions (Eyes Open, and Eyes Closed) at Pre-Assessment

**Table A3:** Correlations between age and FFT frequency band activity at pre assessment—non-ADHD <16 years (n = 25).

	Eyes Open			Eyes Closed		
Electrode Location	Fz	Cz	Pz	Fz	Cz	Pz
**Beta (μV) × age (years)**	−0.609 **	−0.456 *	−0.475 *	−0.492 *	−0.415 *	−0.330
**alpha (μV) × age (years)**	−0.410 *	−0.335	−0.276	−0.201	−0.189	−0.366
**theta (μV) × age (years)**	−0.478 *	−0.249	−0.449 *	−0.467 *	−0.111	−0.409 *
**delta (μV) × age (years)**	−0.566 **	−0.414 *	−0.548 **	−0.543 **	−0.373	−0.508 **

Frequency band widths: Beta (12.5–30 Hz), alpha (7.5–12.5 Hz), theta (3.5–7.5 Hz), and delta (0.5–3.5 Hz). Pearson product-moment correlations are displayed. ** *p* ≤ 0.001, * *p* ≤ 0.01.

**Table A4:** Correlations between age and FFT frequency band activity at pre assessment—ADHD <16 years (n = 150).

	Eyes Open			Eyes Closed		
Electrode Location	Fz	Cz	Pz	Fz	Cz	Pz
**beta (μV) × age (years)**	0.093	0.044	−0.006	−0.075	−0.108	−0.108
**alpha (μV) × age (years)**	−0.038	−0.122	−0.000	−0.091	−0.163	−0.236 **
**theta (μV) × age (years)**	−0.260 **	−0.276 **	−0.240 **	−0.491 **	−0.476 **	−0.353 **
**delta (μV) × age (years)**	−0.126	−0.232 **	−0.243 **	−0.289 **	−0.381 **	−0.202 *

Frequency band widths: beta (12.5–30 Hz), alpha (7.5–12.5 Hz), theta (3.5–7.5 Hz), and delta (0.5–3.5 Hz). Pearson product-moment correlations are displayed. ** *p* ≤ 0.001, * *p* ≤ 0.01.

## Appendix E

**Table A5:** Results from stepwise regression models for eyes open alpha activity at electrode Fz.

		Unstandardized Coefficients		Standardized Coefficients					
Step	Predictor	B	SE	β	*p*	*R* ^2^	Δ*R*^2^	F	*p*
**1**	Age	−0.005	0.001	−0.291	<0.0001	0.085	0.085	200.23	<0.0001
**2**	Age Inattention Hyp/imp	−0.005 0.013 0.010	0.001 0.010 0.010	−0.301 0.100 0.076	<0.0001 0.196 0.321	0.109	0.024	8.83	<0.0001
**3**	Age Inattention Hyp/imp Data quality (artifact-free segments in %)	−0.004 0.011 0.006 −0.001	0.001 0.010 0.010 0.000	−0.273 0.083 0.046 −0.213	<0.0001 0.273 0.549 0.001	0.152	0.043	9.66	<0.0001

N = 220. Hyp/imp = Hyperactivity/impulsivity. SE = standard error of B.

**Table A6:** Results from stepwise regression models for eyes open alpha activity at electrode Cz.

		Unstandardized Coefficients		Standardized Coefficients					
Step	Predictor	B	SE	β	*p*	*R* ^2^	Δ*R*^2^	F	*p*
**1**	Age	−0.007	0.001	−0.295	<0.0001	0.087	0.087	200.94	<0.0001
**2**	Age Inattention Hyp/imp	−0.007 0.030 −0.028	0.001 0.014 0.015	−0.313 0.163 −0.147	<0.0001 0.036 0.057	0.109	0.022	8.84	<0.0001
**3**	Age Inattention Hyp/imp Data quality (artifact-free segments in %)	−0.006 0.028 −0.032 −0.001	0.001 0.014 0.014 0.000	−0.291 0.150 −0.172 −0.168	<0.0001 0.052 0.026 0.011	0.135	0.027	8.46	<0.0001

N = 220. Hyp/imp = Hyperactivity/impulsivity. SE = standard error of B.

**Table A7:** Results from stepwise regression models for eyes open alpha activity at electrode Pz.

		Unstandardized Coefficients		Standardized Coefficients					
Step	Predictor	B	SE	β	*p*	*R* ^2^	Δ*R*^2^	F	*p*
**1**	Age	−0.007	0.003	−0.156	0.020	0.024	0.024	50.49	0.020
**2**	Age Inattention Hyp/imp	−0.007 0.037 0.017	0.003 0.029 0.029	−0.167 0.102 0.046	0.014 0.203 0.567	0.042	0.018	3.18	0.025
**3**	Age Inattention Hyp/imp Data quality (artifact-free segments in %)	−0.007 0.034 0.010 −0.001	0.003 0.029 0.030 0.001	−0.151 0.092 0.028 −0.125	0.026 0.249 0.730 0.067	0.057	0.015	3.25	0.013

N = 220. Hyp/imp = Hyperactivity/impulsivity. SE = standard error of B.

**Table A8:** Results from stepwise regression models for eyes open beta activity at electrode Fz.

		Unstandardized Coefficients		Standardized Coefficients					
Step	Predictor	B	SE	β	*p*	*R* ^2^	Δ*R*^2^	F	*p*
**1**	Age	−0.001	0.000	−0.155	0.021	0.024	0.024	5.38	0.021
**2**	Age Inattention Hyp/imp	−0.001 0.004 0.003	0.000 0.003 0.003	−0.164 0.091 0.086	0.015 0.257 0.282	0.048	0.024	3.66	0.013
**3**	Age Inattention Hyp/imp Data quality (artifact-free segments in %)	−0.001 0.003 0.002 0.000	0.000 0.003 0.003 0.000	−0.139 0.075 0.058 −0.192	0.037 0.339 0.463 0.005	0.083	0.035	4.88	0.001

N = 220. Hyp/imp = Hyperactivity/impulsivity. SE = standard error of B.

**Table A9:** Results from stepwise regression models for eyes open beta activity at electrode Cz.

		Unstandardized Coefficients		Standardized Coefficients					
Step	Predictor	B	SE	β	*p*	*R* ^2^	Δ*R*^2^	F	*p*
**1**	Age	0.000	0.000	−0.092	0.173	0.008	0.008	1.87	0.173
**2**	Age Inattention Hyp/imp	−0.001 0.005 −0.005	0.000 0.004 0.004	−0.104 0.111 −0.109	0.127 0.174 0.177	0.019	0.011	1.42	0.239
**3**	Age Inattention Hyp/imp Data quality (artifact-free segments in %)	0.000 0.004 −0.007 0.000	0.000 0.003 0.003 0.000	−0.066 0.087 −0.152 −0.290	0.313 0.265 0.054 0.000	0.099	0.080	5.93	<0.0001

N = 220. Hyp/imp = Hyperactivity/impulsivity. SE = standard error of B.

**Table A10:** Results from stepwise regression models for eyes open beta activity at electrode Pz.

		Unstandardized Coefficients		Standardized Coefficients					
Step	Predictor	B	SE	β	*p*	*R* ^2^	Δ*R*^2^	F	*p*
**1**	Age	−0.001	0.001	−0.059	0.384	0.003	0.003	0.76	0.384
**2**	Age Inattention Hyp/imp	−0.001 0.008 0.011	0.001 0.011 0.011	−0.065 0.061 0.078	0.341 0.456 0.333	0.018	0.015	1.36	0.256
**3**	Age Inattention Hyp/imp Data quality (artifact-free segments in %)	−0.001 0.007 0.008 −0.001	0.001 0.011 0.011 0.000	−0.048 0.050 0.060 −0.127	0.479 0.535 0.460 0.066	0.034	0.015	1.89	0.114

N = 220. Hyp/imp = Hyperactivity/impulsivity. SE = standard error of B.

**Table A11:** Results from stepwise regression models for eyes open theta activity at electrode Fz.

		Unstandardized Coefficients		Standardized Coefficients					
Step	Predictor	B	SE	β	*p*	*R* ^2^	Δ*R*^2^	F	*p*
**1**	Age	−0.015	0.002	−0.480	<0.0001	0.230	0.230	65.51	<0.0001
**2**	Age Inattention Hyp/imp	−0.015 0.014 0.015	0.002 0.019 0.019	−0.485 0.053 0.055	<0.0001 0.462 0.439	0.239	0.009	22.74	<0.0001
**3**	Age Inattention Hyp/imp Data quality (artifact-free segments in %)	−0.014 0.010 0.007 −0.002	0.002 0.018 0.019 0.000	−0.460 0.037 0.027 −0.193	<0.0001 0.597 0.700 0.001	0.274	0.035	20.43	<0.0001

N = 220. Hyp/imp = Hyperactivity/impulsivity. SE = standard error of B.

**Table A12:** Results from stepwise regression models for eyes open theta activity at electrode Cz.

		Unstandardized Coefficients		Standardized Coefficients					
Step	Predictor	B	SE	β	*p*	*R* ^2^	Δ*R*^2^	F	*p*
**1**	Age	−0.016	0.002	−0.456	<0.0001	0.208	0.208	57.59	<0.0001
**2**	Age Inattention Hyp/imp	−0.017 0.012 −0.029	0.002 0.022 0.022	−0.461 0.039 −0.095	<0.0001 0.587 0.191	0.215	0.006	19.77	<0.0001
**3**	Age Inattention Hyp/imp Data quality (artifact-free segments in %)	−0.016 0.008 −0.037 −0.002	0.002 0.022 0.022 0.001	−0.438 0.025 −0.120 −0.174	<0.0001 0.723 0.095 0.005	0.243	0.029	17.36	<0.0001

N = 220. Hyp/imp = Hyperactivity/impulsivity. SE = standard error of B.

**Table A13:** Results from stepwise regression models for eyes open theta activity at electrode Pz.

		Unstandardized Coefficients		Standardized Coefficients					
Step	Predictor	B	SE	β	*p*	*R* ^2^	Δ*R*^2^	F	*p*
**1**	Age	−0.017	0.003	−0.325	<0.0001	0.106	0.106	25.93	<0.0001
**2**	Age Inattention Hyp/imp	−0.017 0.023 0.033	0.003 0.033 0.033	−0.331 0.054 0.075	<0.0001 0.487 0.327	0.119	0.013	9.75	<0.0001
**3**	Age Inattention Hyp/imp Data quality (artifact-free segments in %)	−0.016 0.018 0.024 −0.002	0.003 0.033 0.033 0.001	−0.314 0.043 0.056 −0.130	<0.0001 0.574 0.464 0.047	0.135	0.016	8.42	<0.0001

N = 220. Hyp/imp = Hyperactivity/impulsivity. SE = standard error of B.

**Table A14:** Results from stepwise regression models for eyes open delta activity at electrode Fz.

		Unstandardized Coefficients		Standardized Coefficients					
Step	Predictor	B	SE	β	*p*	*R* ^2^	Δ*R*^2^	F	*p*
**1**	Age	−0.061	0.009	−0.415	<0.0001	0.172	0.172	44.98	<0.0001
**2**	Age Inattention Hyp/imp	−0.061 0.021 0.104	0.009 0.092 0.093	−0.416 0.017 0.083	<0.0001 0.820 0.266	0.181	0.009	15.78	<0.0001
**3**	Age Inattention Hyp/imp Data quality (artifact-free segments in %)	−0.057 0.005 0.053 −0.009	0.009 0.089 0.091 0.002	−0.389 0.004 0.042 −0.233	<0.0001 0.954 0.565 < 0.0001	0.233	0.051	16.13	<0.0001

N = 217. Hyp/imp = Hyperactivity/impulsivity. SE = standard error of B.

**Table A15:** Results from stepwise regression models for eyes open delta activity at electrode Cz.

		Unstandardized Coefficients		Standardized Coefficients					
Step	Predictor	B	SE	β	*p*	*R* ^2^	Δ*R*^2^	F	*p*
**1**	Age	−0.065	0.010	−0.415	<0.0001	0.172	0.172	45.34	<0.0001
**2**	Age Inattention Hyp/imp	−0.066 0.028 −0.061	0.010 0.098 0.099	−0.418 0.022 −0.046	<0.0001 0.774 0.538	0.174	0.001	15.13	<0.0001
**3**	Age Inattention Hyp/imp Data quality (artifact-free segments in %)	−0.061 0.003 −0.112 −0.010	0.010 0.095 0.097 0.003	−0.387 0.002 −0.084 −0.251	<0.0001 0.976 0.249 < 0.0001	0.233	0.059	16.34	<0.0001

N = 219. Hyp/imp = Hyperactivity/impulsivity. SE = standard error of B.

**Table A16:** Results from stepwise regression models for eyes open delta activity at electrode Pz.

		Unstandardized Coefficients		Standardized Coefficients					
Step	Predictor	B	SE	β	*p*	*R* ^2^	Δ*R*^2^	F	*p*
**1**	Age	−0.071	0.011	−0.410	<0.0001	0.168	0.168	44.25	<0.0001
**2**	Age Inattention Hyp/imp	−0.072 0.104 −0.016	0.011 0.108 0.108	−0.417 0.072 −0.011	<0.0001 0.337 0.886	0.172	0.004	15.107	<0.0001
**3**	Age Inattention Hyp/imp Data quality (artifact-free segments in %)	−0.066 0.074 −0.070 −0.102	0.010 0.104 0.105 0.003	−0.384 0.051 −0.048 −0.258	<0.0001 0.479 0.504 < 0.0001	0.236	0.063	16.64	<0.0001

N = 220. Hyp/imp = Hyperactivity/impulsivity. SE = standard error of B.

**Table A17:** Results from stepwise regression models for eyes closed alpha activity at electrode Fz.

		Unstandardized Coefficients		Standardized Coefficients					
Step	Predictor	B	SE	β	*p*	*R* ^2^	Δ*R*^2^	F	*p*
**1**	Age	−0.006	0.002	−0.209	0.002	0.044	0.044	9.50	0.002
**2**	Age Inattention Hyp/imp	−0.006 0.012 0.023	0.002 0.017 0.018	−0.216 0.057 0.105	0.002 0.483 0.196	0.065	0.021	4.72	0.003
**3**	Age Inattention Hyp/imp Data quality (artifact-free segments in %)	−0.005 0.011 0.018 −0.001	0.002 0.017 0.018 0.000	−0.185 0.053 0.079 −0.111	0.010 0.515 0.339 0.128	0.075	0.011	4.15	0.003

N = 208. Hyp/imp = Hyperactivity/impulsivity. SE = standard error of B.

**Table A18:** Results from stepwise regression models for eyes closed alpha activity at electrode Cz.

		Unstandardized Coefficients		Standardized Coefficients					
Step	Predictor	B	SE	β	*p*	*R* ^2^	Δ*R*^2^	F	*p*
**1**	Age	−0.009	0.002	−0.306	<0.0001	0.094	0.094	21.37	<0.0001
**2**	Age Inattention Hyp/imp	−0.010 0.034 −0.012	0.002 0.019 0.020	−0.324 0.144 −0.050	<0.0001 0.072 0.528	0.109	0.015	8.33	<0.0001
**3**	Age Inattention Hyp/imp Data quality (artifact-free segments in %)	−0.009 0.033 −0.019 −0.001	0.002 0.019 0.020 0.001	−0.292 0.140 −0.077 −0.116	<0.0001 0.080 0.339 0.102	0.120	0.012	6.97	<0.0001

N = 208. Hyp/imp = Hyperactivity/impulsivity. SE = standard error of B.

**Table A19:** Results from stepwise regression models for eyes closed alpha activity at electrode Pz.

		Unstandardized Coefficients		Standardized Coefficients					
Step	Predictor	B	SE	β	*p*	*R* ^2^	Δ*R*^2^	F	*p*
**1**	Age	−0.022	0.006	−0.268	<0.0001	0.072	0.072	16.03	<0.0001
**2**	Age Inattention Hyp/imp	−0.023 0.043 0.044	0.006 0.054 0.055	−0.276 0.065 −0.064	<0.0001 0.423 0.427	0.085	0.013	6.31	<0.0001
**3**	Age Inattention Hyp/imp Data quality (artifact-free segments in %)	−0.021 0.041 0.031 −0.002	0.006 0.054 0.056 0.001	−0.254 0.062 0.046 −0.077	<0.0001 0.444 0.578 0.285	0.090	0.005	5.03	0.001

N = 208. Hyp/imp = Hyperactivity/impulsivity. SE = standard error of B.

**Table A20:** Results from stepwise regression models for eyes closed beta activity at electrode Fz.

		Unstandardized Coefficients		Standardized Coefficients					
Step	Predictor	B	SE	β	*p*	*R* ^2^	Δ*R*^2^	F	*p*
**1**	Age	−0.001	0.000	−0.151	0.029	0.023	0.023	14.81	0.029
**2**	Age Inattention Hyp/imp	−0.001 0.004 0.001	0.000 0.003 0.003	−0.164 0.112 0.014	0.019 0.176 0.867	0.037	0.014	2.63	0.052
**3**	Age Inattention Hyp/imp Data quality (artifact-free segments in %)	−0.001 0.004 −0.001 0.000	0.000 0.003 0.003 0.000	−0.117 0.106 −0.027 −0.173	0.104 0.198 0.749 0.019	0.063	0.026	3.41	0.010

N = 208. Hyp/imp = Hyperactivity/impulsivity. SE = standard error of B.

**Table A21:** Results from stepwise regression models for eyes closed beta activity at electrode Cz.

		Unstandardized Coefficients		Standardized Coefficients					
Step	Predictor	B	SE	β	*p*	*R* ^2^	Δ*R*^2^	F	*p*
**1**	Age	0.000	0.000	−0.123	0.076	0.015	0.015	3.19	0.076
**2**	Age Inattention Hyp/imp	−0.001 0.004 −0.002	0.000 0.002 0.002	−0.141 0.137 −0.072	0.045 0.102 0.385	0.028	0.013	1.97	0.120
**3**	Age Inattention Hyp/imp Data quality (artifact-free segments in %)	0.000 0.004 −0.004 0.000	0.000 0.002 0.002 0.000	−0.060 0.125 −0.140 −0.293	0.390 0.120 0.086 <0.0001	0.102	0.074	5.77	<0.0001

N = 208. Hyp/imp = Hyperactivity/impulsivity. SE = standard error of B.

**Table A22:** Results from stepwise regression models for eyes closed beta activity at electrode Pz.

		Unstandardized Coefficients		Standardized Coefficients					
Step	Predictor	B	SE	β	*p*	*R* ^2^	Δ*R*^2^	F	*p*
**1**	Age	−0.001	0.000	−0.170	0.014	0.029	0.029	6.17	0.014
**2**	Age Inattention Hyp/imp	−0.001 0.006 −0.005	0.000 0.003 0.003	−0.190 0.156 −0.121	0.006 0.060 0.141	0.047	0.018	3.36	0.020
**3**	Age Inattention Hyp/imp Data quality (artifact-free segments in %)	0.000 0.005 −0.007 0.000	0.000 0.003 0.003 0.000	−0.106 0.144 −0.192 −0.306	0.125 0.070 0.017 <0.0001	0.128	0.081	7.46	<0.0001

N = 208. Hyp/imp = Hyperactivity/impulsivity. SE = standard error of B.

**Table A23:** Results from stepwise regression models for eyes closed theta activity at electrode Fz.

		Unstandardized Coefficients		Standardized Coefficients					
Step	Predictor	B	SE	β	*p*	*R* ^2^	Δ*R*^2^	F	*p*
**1**	Age	−0.017	0.002	−0.520	<0.0001	0.271	0.271	76.82	<0.0001
**2**	Age Inattention Hyp/imp	−0.016 −0.013 0.010	0.002 0.018 0.019	−0.514 −0.051 0.037	<0.0001 0.482 0.604	0.273	0.002	25.60	<0.0001
**3**	Age Inattention Hyp/imp Data quality (artifact-free segments in %)	−0.014 −0.015 −0.005 −0.002	0.002 0.018 0.019 0.000	−0.446 −0.060 −0.020 −0.246	<0.0001 0.388 0.772 <0.0001	0.325	0.052	24.52	<0.0001

N = 208. Hyp/imp = Hyperactivity/impulsivity. SE = standard error of B.

**Table A24:** Results from stepwise regression models for eyes closed theta activity at electrode Cz.

		Unstandardized Coefficients		Standardized Coefficients					
Step	Predictor	B	SE	β	*p*	*R* ^2^	Δ*R*^2^	F	*p*
**1**	Age	−0.018	0.002	−0.472	<0.0001	0.223	0.223	59.48	<0.0001
**2**	Age Inattention Hyp/imp	−0.018 −0.008 0.011	0.002 0.023 0.024	−0.469 −0.027 0.035	<0.0001 0.718 0.640	0.224	0.001	19.74	<0.0001
**3**	Age Inattention Hyp/imp Data quality (artifact-free segments in %)	−0.016 −0.011 −0.007 −0.002	0.002 0.022 0.024 0.001	−0.403 −0.036 −0.021 −0.238	<0.0001 0.618 0.772 <0.0001	0.273	0.049	19.15	<0.0001

N = 208. Hyp/imp = Hyperactivity/impulsivity. SE = standard error of B.

**Table A25:** Results from stepwise regression models for eyes closed theta activity at electrode Pz.

		Unstandardized Coefficients		Standardized Coefficients					
Step	Predictor	B	SE	β	*p*	*R* ^2^	Δ*R*^2^	F	*p*
**1**	Age	−0.027	0.005	−0.383	<0.0001	0.147	0.147	35.67	<0.0001
**2**	Age Inattention Hyp/imp	−0.027 −0.014 0.049	0.005 0.044 0.045	−0.380 −0.024 0.084	<0.0001 0.757 0.279	0.152	0.005	12.28	<0.0001
**3**	Age Inattention Hyp/imp Data quality (artifact-free segments in %)	−0.023 −0.018 0.020 −0.004	0.005 0.043 0.045 0.001	−0.321 −0.032 0.034 −0.213	<0.0001 0.671 0.661 0.002	0.191	0.039	12.07	<0.0001

N = 208. Hyp/imp = Hyperactivity/impulsivity. SE = standard error of B.

**Table A26:** Results from stepwise regression models for eyes closed delta activity at electrode Fz.

		Unstandardized Coefficients		Standardized Coefficients					
Step	Predictor	B	SE	β	*p*	*R* ^2^	Δ*R*^2^	F	*p*
**1**	Age	−0.063	0.010	−0.383	<0.0001	0.165	0.165	40.67	<0.0001
**2**	Age Inattention Hyp/imp	−0.062 −0.031 0.025	0.010 0.096 0.099	−0.380 −0.024 0.084	<0.0001 0.748 0.799	0.165	0.000	13.47	<0.0001
**3**	Age Inattention Hyp/imp Data quality (artifact-free segments in %)	−0.049 −0.047 −0.064 −0.012	0.010 0.091 0.096 0.003	−0.321 −0.032 0.034 −0.213	<0.0001 0.604 0.504 <0.0001	0.247	0.082	16.66	<0.0001

N = 207. Hyp/imp = Hyperactivity/impulsivity. SE = standard error of B.

**Table A27:** Results from stepwise regression models for eyes closed delta activity at electrode Fz.

		Unstandardized Coefficients		Standardized Coefficients					
Step	Predictor	B	SE	β	*p*	*R* ^2^	Δ*R*^2^	F	*p*
**1**	Age	−0.057	0.008	−0.451	<0.0001	0.203	0.203	52.61	<0.0001
**2**	Age Inattention Hyp/imp	−0.056 −0.025 0.068	0.008 0.077 0.079	−0.448 −0.024 0.065	<0.0001 0.747 0.389	0.206	0.003	17.69	<0.0001
**3**	Age Inattention Hyp/imp Data quality (artifact-free segments in %)	−0.046 −0.042 0.000 −0.010	0.008 0.073 0.077 0.002	−0.365 −0.041 0.000 −0.301	<0.0001 0.570 0.995 <0.0001	0.285	0.078	20.21	<0.0001

N = 207. Hyp/imp = Hyperactivity/impulsivity. SE = standard error of B.

**Table A28:** Results from stepwise regression models for eyes closed delta activity at electrode Pz.

		Unstandardized Coefficients		Standardized Coefficients					
Step	Predictor	B	SE	β	*p*	*R* ^2^	Δ*R*^2^	F	*p*
**1**	Age	−0.077	0.013	−0.382	<0.0001	0.146	0.146	35.43	<0.0001
**2**	Age Inattention Hyp/imp	−0.077 0.041 −0.032	0.013 0.125 0.129	−0.386 0.026 −0.019	<0.0001 0.742 0.802	0.147	0.000	11.74	<0.0001
**3**	Age Inattention Hyp/imp Data quality (artifact-free segments in %)	−0.060 0.022 −0.153 −0.015	0.013 0.119 0.126 0.003	−0.301 0.014 −0.091 −0.309	<0.0001 0.853 0.226 <0.0001	0.229	0.082	15.12	<0.0001

N = 208. Hyp/imp = Hyperactivity/impulsivity. SE = standard error of B.
